# Synthesis of Two-Dimensional C–C Bonded Truxene-Based Covalent Organic Frameworks by Irreversible Brønsted Acid-Catalyzed Aldol Cyclotrimerization

**DOI:** 10.34133/2021/9790705

**Published:** 2021-09-03

**Authors:** Qingsong Zhang, Yunlong Sun, Haijing Li, Kun Tang, Yu-Wu Zhong, Dong Wang, Yunlong Guo, Yunqi Liu

**Affiliations:** ^1^Beijing National Laboratory for Molecular Sciences, Institute of Chemistry, Chinese Academy of Sciences, Beijing 100190, China; ^2^University of Chinese Academy of Sciences, Beijing 100049, China; ^3^Beijing Synchrotron Radiation Facility, Institute of High Energy Physics, Chinese Academy of Sciences, Beijing 100049, China

## Abstract

The synthesis of new C–C bonded two-dimensional (2D) covalent organic frameworks (COFs) is highly desirable. Here, a simple but effective synthetic strategy has been developed using an irreversible Brønsted acid-catalyzed aldol cyclotrimerization reaction by virtue of truxene as a linkage. Nonolefin C–C bonded 2D truxene-based covalent organic frameworks (Tru-COFs) were constructed by polymerization of 1,3,5-triindanonebenzene (TDB). The structure formation was confirmed by wide-angle X-ray scattering, Fourier-transform infrared spectroscopy, and solid-state ^13^C CP/MAS NMR. The results showed that the Tru-COFs were porous (645 m^2^/g) and chemically stable. Benzyl methylene in conjugated Tru-COFs more effectively produced photoinduced radicals than the model truxene compound. Due to the radical photoresponsiveness, Tru-COFs were efficient catalysts for photocatalytic oxidation of sulfides. We expect that this will provide a new synthetic methodology to obtain C–C bonded functional 2D COFs.

## 1. Introduction

Covalent organic frameworks (COFs) are two- or three-dimensional porous crystalline materials. Since the first COFs were reported in 2005, many COFs with different linkages have been obtained using a variety of synthetic strategies, and the resulting materials have shown applications in gas storage/separation, energy storage, semiconductor devices, and photocatalysis [1–8]. However, most reported COFs were synthesized by reversible reactions and were linked through bonds such as borate esters and imines, which are chemically unstable. The low degree of in-plane electron delocalization of the COFs is another bottleneck for their use as photoelectric materials [9]. An ideal synthetic strategy would form COFs with irreversible C–C bonds that possess higher chemical stability and probably greater electron delocalization, for example, the COF with sp^2^-bonded skeleton reported by Zhuang and coworkers [10].

COFs synthesized through reversible reactions contain self-healing crystal defects, making it very difficult to prepare crystalline COFs via irreversible reactions. To our knowledge, there are relatively few cases for directly connecting two aromatic blocks by C–C bonds to form crystalline COFs, including the Ullmann reaction [11], Glaser coupling [12, 13], liquid-liquid interface polymerization by Suzuki coupling [14], and bulk crystal synthesis by the Knoevenagel condensation and aldol condensation [10, 15–21]. Despite these methods, it is still challenging to find viable irreversible reactions to construct two-dimensional (2D) COFs using C–C bonds.

**Scheme 1:**
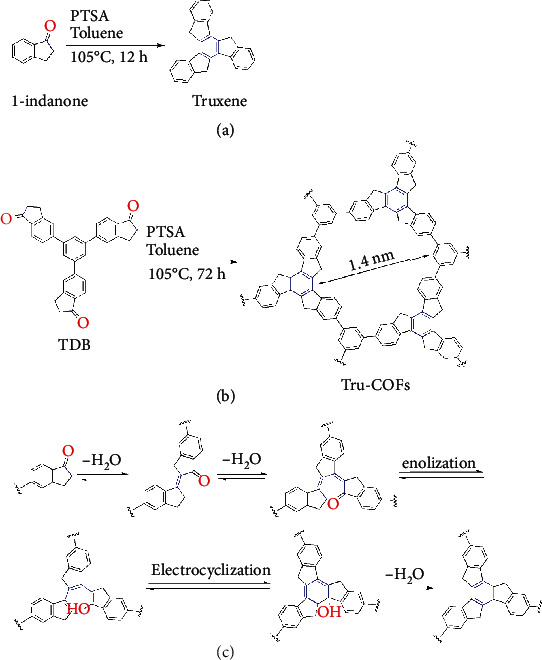
(a) Model compound synthesis; (b) synthesis of Tru-COFs; (c) proposed mechanism of truxene linkage formation.

Brønsted acid-catalyzed aldol cyclotrimerization (BAAC) is a simple self-condensation reaction with only one substrate (Scheme 1(a)). Compared with other methods to synthesize 2D COFs with C–C bonds, especially the Knoevenagel condensation, the BAAC reaction can irreversibly form conjugated polymers, even without olefins. Therefore, COFs synthesized by BAAC reaction should be chemically stable.

Here, we report the first irreversible BAAC reaction to form C–C bonded 2D Tru-COFs using truxene as linkages (Scheme 1(b)). The new material possessed a high surface area, a fully sp^2^-bonded carbon skeleton, and high chemical stability in 9 M HCl and 9 M NaOH. Comparing with borate linkage and imine linkage, the sp^2^-bonded carbon skeleton is more suitable for the in-plane electron delocalization [10, 15]. Moreover, the benzyl methylene moieties in the conjugated Tru-COF structure showed more effective photoinduced radical formation than the model truxene compound under Xe lamp irradiation. Under the same lighting conditions, the intensity of the electron spin resonance (ESR) signal of Tru-COFs was more than twenty times higher than that of truxene. We also demonstrated the use of Tru-COFs as a catalyst for photocatalytic oxidation of sulfides.

BAAC reactions were an efficient reaction to synthesize truxene and its derivatives [22–24]; however, it was difficult to obtain the proper reaction conditions for preparation of crystalline COFs, and most of the efforts can only obtain amorphous products. By screening the reaction conditions, Tru-COFs were finally crystallized in toluene and catalyzed by *p*-toluenesulfonic acid monohydrate (PTSA).

## 2. Result and Discussion

As shown in Table 1 and Table S1, we screened various solvents and reaction temperatures. At 105°C, polymers were obtained using *o*-dichlorobenzene or toluene as the solvent. Most importantly, when toluene was used as the solvent, moderate crystalline polymers can also be obtained. In addition, when 1.0 equiv. PTSA was used, the isolated yield of Tru-COFs was 74.8%, and the BET surface area was 645 m^2^/g (entry 7, Table S1). The best condition was also used to synthesize the model truxene compound (Scheme 1(a)).

The chemical structure of Tru-COFs was then confirmed using FT-IR spectroscopy (Figure S1). The peak at 1697 cm^–1^ assigned to the C=O stretch in the precursor nearly disappeared, and two new peaks appeared at 1711 cm^–1^ and 1683 cm^–1^, which belonged to the C=O stretch in the intermediate formed during Tru-COF synthesis. Moreover, these two peaks showed lower absorbance intensities. During cyclomerization (Scheme 1(c)), indanone formed a dimer intermediate and then a trimer intermediate structure, followed by the reversible removal of two H_2_O molecules. According to a previous report, the trimer intermediate forms a six-member ring, followed by the formation of a truxene structure after irreversible H_2_O elimination [25, 26].

Solid-state ^13^C CP/MAS NMR analysis of the Tru-COFs and the TDB precursor indicates that cyclomerization occurred (Figure S2). In the TDB spectrum, the peak at 206 ppm belonged to the carbonyl carbons, while the peaks at 37 ppm and 25 ppm belonged to alkyl carbons. In the Tru-COF spectrum, the peak at 206 ppm nearly disappeared, and only one alkyl carbon peak was observed at 32 ppm, demonstrating cyclomerization and the formation of a truxene structure.

**Figure 1:**
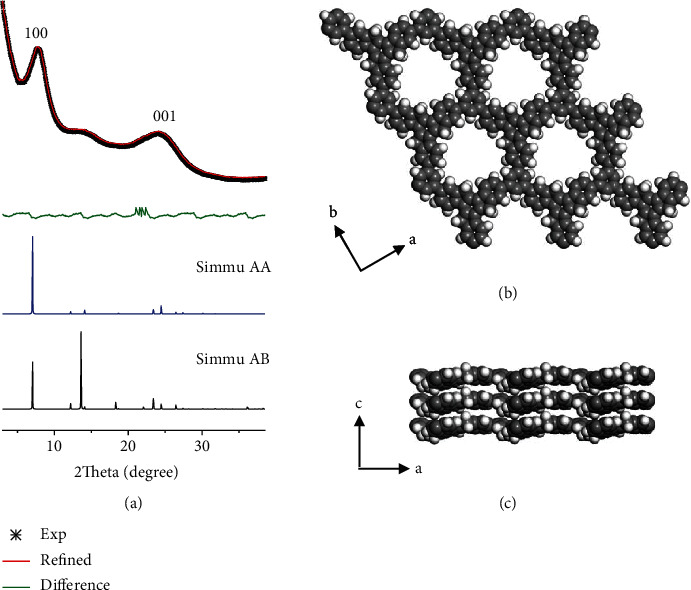
(a) Experimental WAXS patterns of Tru-COFs, Pawley-refined pattern, the difference between the experimental and calculated AA stacking pattern, calculated patterns for AB stacking and AA stacking. (b) Top view and (c) side view of Tru-COFs in AA stacking. Color code: H: white; C: gray.

The crystallinity of Tru-COFs was confirmed by WAXS and featured a main peak at 7.7° (100). Materials Studio 8.0 was used to build the 2D Tru-COF structure models (Figure 1, Figure S3, and Table S2). The AA stacking model matched the experimental data, and the (100) peak was around 7.1°. There was a certain deviation between the simulated value and the experimental value (about 0.6°). This situation also exists in covalent triazine frameworks and phenazine-linked CS-COF [27–29]. Pawley profile refinement of the model against the experimental pattern yielded a unit cell of *a* = *b* = 14.4 Å, *c* = 3.48 Å, *α* = *β* = 90°, and *γ* = 120° with good agreement factors (Rwp = 7.41%, Rp = 3.79%).

**Figure 2:**
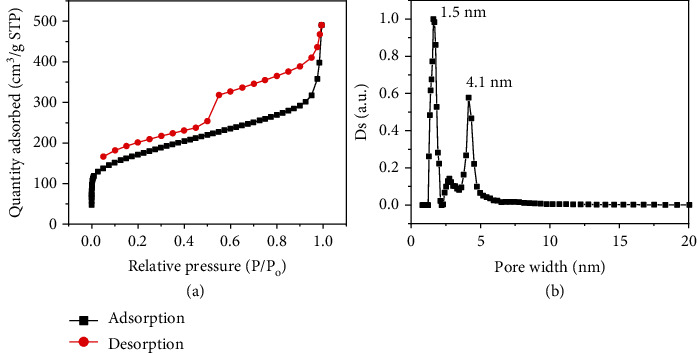
(a) N_2_ adsorption/desorption isotherms at 77 K and (b) pore size distribution of the Tru-COF synthesized with 0.6 equiv. PTSA.

The permanent porosity of Tru-COFs was measured by N_2_ adsorption at 77 K. As shown in Table S1, Figure 2, and Figure S4, the porosity could be tuned by changing the amount of PTSA during synthesis. When the amount of PTSA was 0.6 equiv., the reaction provided crystalline Tru-COFs with the highest BET surface area of 657.8 m^2^/g in 38.7% isolated yield (entry 7, Table S1). Further increasing the amount of PTSA to 1.0 equiv. increased the COF yield up to 74.8% (entry 9, Table S1), but it did not increase the BET surface area, that is, 645 m^2^/g. The theoretical surface area of Tru-COFs calculated using Zeo++ code was 984.9 m^2^/g [30]. Compared with the theoretical value, the experimental value (645 m^2^/g) was obviously smaller. We speculate that some irregular pores were irreversibly formed during the reaction, and there are some unreacted defects inside, which might have resulted in a relatively smaller surface area. Furthermore, nonlocal density functional theory calculations reveal that Tru-COFs show a narrow pore size distribution, with a peak maximum at 1.5 nm (Figure 2(b)), the data of which agree well with the calculated pore sizes (1.4 nm) based on the AA stacking models (Scheme 1(b) and Figure 1(b)).

Tru-COFs were found to be thermally stable up to 450°C by thermogravimetric analysis (Figure S5). The Tru-COFs also showed high chemical stability. After immersing the COF samples in 9 M HCl and 9 M NaOH solution for 24 h, the samples retained their original wide-angle X-ray scattering (WAXS) profiles and the original FT-IR spectra (Figures S6 and S7). The BET surface area of Tru-COFs decreased (579 m^2^/g) after immersion in 9 M NaOH and showed almost no change after immersion in 9 M HCl (680 m^2^/g) (Figure S8).

**Figure 3:**
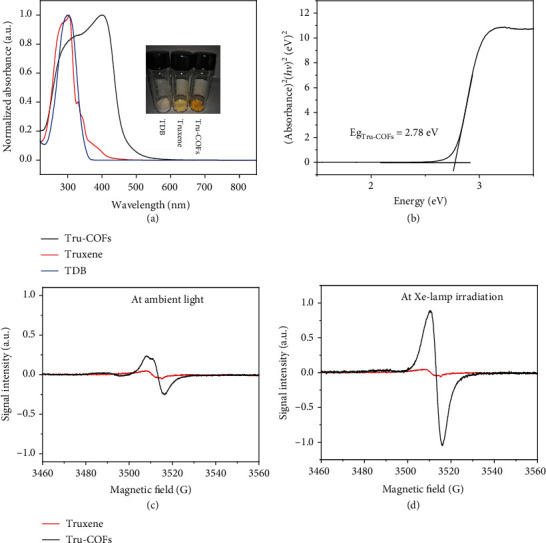
(a) Solid-state UV-Vis diffuse reflectance spectrum. (b) The plot of the Kubelka-Munk function to determine the band gap of Tru-COFs. (c) Solid ESR spectrum of the model truxene compound and Tru-COFs showing the weak signal intensity in dark ambient light conditions. (d) Solid-state ESR spectra of truxene and Tru-COFs after Xe lamp irradiation for 120 s.

The photophysical properties of the Tru-COFs are important for their potential applications (Figure 3). In the solid-state ultraviolet-visible (UV-Vis) spectra, the Soret band of Tru-COFs showed a red shift of 166 nm compared with truxene, demonstrating the formation of a *π*-conjugated system in the material (Figure 3(a)). And the optical band gap of the Tru-COFs was estimated to be 2.78 eV (Figure 3(b)). To determine the highest occupied molecular orbital (HOMO), we performed cyclic voltammetry (Figure S9). The onset oxidation potential was 0.722 V. After referring to the redox potential of ferrocene/ferrocenium, the HOMO energy of Tru-COFs was calculated to be –5.16 eV, and the LUMO energy of Tru-COFs was calculated to be –2.38 eV.

Benzyl methylene acts as an active site in conjugated molecules and can be triggered by light excitation to generate radicals [31]. We performed electron spin resonance (ESR) spectroscopy on the model truxene compound and the Tru-COFs. As shown in Figure S10, the truxene compound showed a weak signal intensity in the dark, which might be due to the absorbance of ambient light. It should be noted that the ambient light intensity was much lower than that of Xe lamp. And the condition of dark reaction refers to the reaction without Xe lamp irradiation. The absorbance of truxene was already saturated because there were almost no changes during Xe lamp irradiation. The weak ESR signal of truxene indicated that truxene was probably a radical photoresponsive structure. With these results, we tested the ESR signal intensity of Tru-COFs. Similarly, the COFs showed a weak signal intensity in the dark reaction (Figure 3(c)). Interestingly, the signal intensity greatly increased and then reached saturation during Xe lamp irradiation (Figure 3(d)). As shown in Figure 3(d), with Xe lamp irradiation, the ESR peak signal intensity of Tru-COFs was twenty times higher than the model truxene compound. When the light source was removed, the signal intensity was gradually attenuated (Figure S10b). The TDB monomer showed no ESR signal. The ESR experiments demonstrated that Tru-COFs was a radical photoresponsive material, possibly due to the stronger electron delocalization than the model truxene compound.

**Figure 4:**
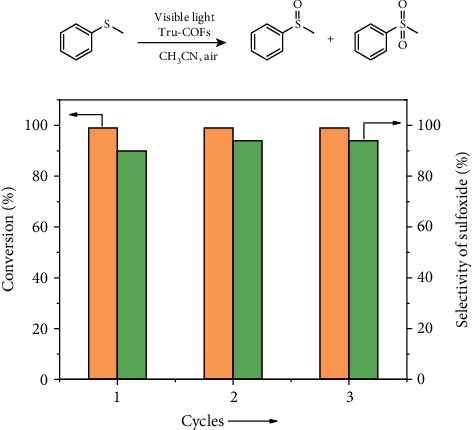
Recyclability of Tru-COFs in sulfate oxidation reaction. Conditions: air (1 atm), 0.25 mmol thioanisole, 5 mg (0.036 mmol) Tru-COFs, 5 mL CH_3_CN, 4 h, 300 W Xe lamp, *λ* > 420 nm, 25°C. The conversion of the reaction and the selectivity of sulfoxide were determined by ^1^H NMR.

Considering the radical photoresponsiveness, chemical stability, suitable HOMO/LOMO energy, and optical band gap of this material, it may display photocatalytic activity. Visible light-driven aerobic oxidation plays a vital role in the environmental and green chemistry, and it is also an important way to synthesize high-value products [32–34]. Here, we found that Tru-COFs showed good performance in photocatalytic oxidation of sulfides. As shown in Table 2, 0.25 mmol thioanisole and 5 mg Tru-COFs were mixed in 5 mL acetonitrile. The reaction was carried out at 25°C and irradiated with 300 W Xe lamp (*λ* > 420 nm) at 1.0 atm air. The conversion of thioanisole reached 100% in 4 h, with selectivity of methyl phenyl sulfoxide up to 90%. The catalyst was centrifuged and washed, and the recycled catalyst showed no obvious deterioration of conversion and selectivity after the 3rd cycle (Figure 4 and Table S3). Furthermore, the photocatalytic activity of Tru-COFs for other sulfide derivates was investigated. As shown in Table 2, Tru-COFs showed good performance for oxidation of various sulfide derivates with high conversion and selectivity. The mechanism of the process may include light-activated e^−^/h^+^ pair generation and separation (Figure S18) [16, 35].

## 3. Conclusion

In summary, using truxene as an irreversible linkage, a new method was developed to synthesize C–C bonded 2D COFs. This method was used to prepare 2D Tru-COFs without an olefin, which possessed permanent porosity and good chemical stability. Due to the production of photoinduced radicals, Tru-COFs can act as an efficient catalyst for visible light-driven photocatalytic oxidation of sulfides.

## 4. Materials and Methods

For the synthesis of Tru-COFs, a Pyrex tube was filled with TDB (187 mg, 0.4 mmol, 1.0 equiv), PTSA (76 mg, 0.4 mmol, 1.0 equiv), and 10 mL dry toluene. The mixture was sonicated for 1 minute. Then, the mixture was degassed through three freeze-pump-thaw cycles; the tube was sealed under vacuum and heated at 105°C for 3 days. The mixture was cooled to room temperature and collected by filtration and washed with water, ethanol, and DCM several times. The tea bag was Soxhlet extracted in DCM for 2 days and dried under vacuum at 180°C for 4 h to afford the powder (140 mg) in 74.8% isolated yield. Anal. Calcd. for C_11_H_6_·H_2_O: C, 84.58; H, 5.16; O, 10.24. Found: C, 84.58; H, 4.94; O, 6.25. It should be pointed out that the water added in the molecular formula may originate from the water absorbed during the sample preparation process, due to the hygroscopicity of the material.

## Figures and Tables

**Table 1:** Optimization of reaction conditions for Brønsted acid-catalyzed aldol cyclomerization of TDB.

Entry	Solvent	*T* (°C)	Product	BET surface area (m^2^/g)
1^a^	1,4-Dioxane	105	No solid	^e^
2^a^	o-Dichlorobenzene	105	Amorphous	^e^
3^a^	Toluene	90	Amorphous	^e^
4^a^	Toluene	105	Amorphous	107
5^b^	Toluene	105	MC^d^	658
6^c^	Toluene	105	MC^d^	643
7^a^	Toluene	105	MC^d^	645

^a^Reaction catalyzed by 1.0 equiv. PTSA. ^b^Reaction catalyzed by 0.6 equiv. PTSA. ^c^Reaction catalyzed by 0.8 equiv. PTSA. ^d^Moderately crystalline. ^e^Not determined.

**Table 2:** Photocatalytic selective oxidation of thioanisole. The reaction was carried out by using 0.25 mmol of substrate, 0.036 mmol of Tru-COFs (the amount is based on the smallest repeating unit of the structure in the polymer), 5 mL CH_3_CN, 300 W Xe lamp, *λ* > 420 nm, 25°C, air (1 atm).

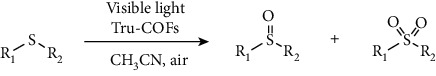				
Entry	Substrate	*T* (h)	Conv (%)	Select (%)
1	R_1_ = Ph R_2_ = CH_3_	4	100	90
2	R_1_ = p‐Me‐Ph R_2_ = CH_3_	4	100	94
3	R_1_ = p‐MeO‐Ph R_2_ = CH_3_	4	100	91
4	R_1_ = p‐Cl‐Ph R_2_ = CH_3_	4	64	99
5	R_1_ = p‐Br‐Ph R_2_ = CH_3_	4	100	93
6	R_1_ = Ph R_2_ = C_2_H_5_	4	97	87

## Data Availability

All data needed in the paper are present in the paper and in the supplementary section. Additional data which are related to this paper may be requested from the authors.

## Supplementary Materials

Supplementary Materials Scheme S1: synthesis of TDB. Scheme S2: synthesis of model compound truxene. Table S1: optimization of reaction conditions for Brønsted acid-catalyzed aldol cyclomerization of TDB. Figure S1: FT-IR spectra of TDB (red), the model compound truxene (green), and Tru-COFs (black). Figure S2: comparison of the solid-state ^13^C NMR (101 MHz) spectrum of Tru-COFs (top) with the model compound truxene (middle) and TDB (bottom). Figure S3: (a) top and (b) side view of the simulated structure of AA stacking and (c) top and (d) side view of the simulated structure of AB stacking. Table S2: unit cell parameters and fractional atomic coordinates for Tru-COF-based AA topology after unit cell correction and Pawley refinement. Figure S4: (a) 77 K N_2_ adsorption and desorption isotherms and (b) pore size distributions of Tru-COF sample synthesized with 0.2 equiv. PTSA. (c) 77 K N_2_ adsorption and desorption isotherms and (d) pore size distributions of Tru-COF sample synthesized with 0.4 equiv. PTSA. (e) 77 K N_2_ adsorption and desorption isotherms and (f) pore size distributions of Tru-COF sample synthesized with 0.6 equiv. PTSA. (g) 77 K N_2_ adsorption and desorption isotherms and (h) pore size distributions of Tru-COF sample synthesized with 0.8 equiv. PTSA. Figure S5: TGA profiles of Tru-COFs. Figure S6: WAXS profiles of Tru-COFs (blue) and recycled Tru-COFs after immersion in 9 M NaOH (black) and 9 M HCl (red) solutions for 24 h, respectively. Figure S7: FT-IR spectra of Tru-COFs (blue) and recycled Tru-COFs after immersion in 9 M NaOH (black) and 9 M HCl (red) solutions for 24 h, respectively. Figure S8: (a) 77 K N_2_ adsorption and desorption isotherms and (b) pore size distributions of Tru-COFs (blue) and recycled Tru-COFs after immersion in 9 M NaOH (black) and 9 M HCl (red) solutions for 24 h, respectively. Figure S9: the CV curves of the Tru-COFs measured in acetonitrile at 298 K. Figure S10: (a) solid ESR spectrum of Tru-COF sample, the ESR peak intensity was changed after Xe lamp irradiation, black line is the dark reaction, red line is the sample irradiated for 40 sec, and green line is the sample irradiated for 120 sec. (b) Solid ESR spectrum of Tru-COF sample, color black stands for the material photo initiated radicals up to saturation, the Xe lamp was then turned off, the ESR experiment was done every 4.3 min once, and the signal intensity was getting weaker obviously. (c) Plot of peak-to-peak height of the ESR signals versus irradiation time. The peak intensity was saturated after 120 sec and decreased after the Xe lamp was turned off. (d) Solid ESR spectrum of model compound truxene showed weak signal intensity in dark reaction for the absorbance of ambient light (black line), and the absorbance was already saturated for there was nothing changed with the Xe lamp irradiation (red line). Figure S11: TEM image of Tru-COFs. Figure S12: TEM image of Tru-COFs. Figure S13: SEM image of Tru-COFs. Table S3: recycle test of Tru-COFs in thioanisole oxidation reaction. Conditions: air (1 atm), 0.25 mmol thioanisole, 5 mg (0.036 mmol) Tru-COFs, 5 mL CH_3_CN, 4 h, 300 W Xe lamp, *λ* > 420 nm, 25°C. The conversion of the reaction and the selectivity of sulfoxide were determined by ^1^H NMR. Figure S14: WAXS profile of Tru-COFs (black) and Tru-COFs after the 1st cycle (red) and Tru-COFs after the 3rd cycle (blue) of photocatalytic oxidation of thioanisole. Figure S15: FT-IR spectra of Tru-COFs (black), Tru-COFs after the 1st cycle (red), and Tru-COFs after the 3rd cycle (blue) of photocatalytic oxidation of thioanisole. Figure S16: SEM image of Tru-COFs after the 1st cycle. Figure S17: SEM image of Tru-COFs after the 3rd cycle. Figure S18: a plausible mechanism for Tru-COFs catalyzed by the visible light-driven selective oxidation of sulfides. Figure S19: (a) schematic of photocatalyzed sacrificial oxygen evolution. Using Na_2_S_2_O_8_ as sacrificial reagent in 10 mM buffer solution, *n*(Na_2_HPO_4_)/*n*(NaH_2_PO_4_) = 6.17, pH = 8. (b) Sacrificial oxygen evolution experiment with 5 mg Tru-COFs as WOC (red line) and without Tru-COFs (black line). Figure S20: WAXS profile of Tru-COFs (blue) and recycled Tru-COFs (yellow) after being used as water oxidation catalyst (WOC) for 4 h. Compared with the as-prepared sample, the WAXS of Tru-COFs (used for 4 h) showed a weaker signal of the (100) peak. Figure S21: FT-IR of Tru-COFs (blue) and recycled Tru-COFs (yellow) after being used as water oxidation catalyst (WOC) for 4 h. The FT-IR spectrum showed almost no change. Figure S22: (a) 77 K N_2_ adsorption and desorption isotherms and (b) pore size distributions of Tru-COFs (blue) and recycled Tru-COFs (yellow) after being used as water oxidation catalyst (WOC) for 4 h. The BET decreased (432 m^2^/g) (Figure S13) compared with the fresh sample, possibly due to a change in the crystallinity. Figure S23: SEM image of Tru-COFs (after 4 h of photocatalytic sacrificial oxidation evolution reaction). Figure S24: TEM image of Tru-COFs (after 4 h of photocatalytic sacrificial oxidation evolution reaction). Figure S25: light-driven water oxidation by Tru-COFs with sodium peroxodisulfate as the sacrificial electron acceptor [7, 8]. Figure S26: powder X-ray diffraction of Tru-COFs (as-synthesized). Figure S27: solid-state ^13^C CP/MAS NMR (101 MHz) spectra of Tru-COFs after 24 h (black) or 72 h (red) polymerization. Figure S28: ^1^H NMR (400 MHz) spectrum of compound TDB in CDCl_3_. Figure S29: ^13^C NMR (101 MHz) spectrum of compound TDB in CDCl_3_. Figure S30: ^1^H NMR (400 MHz) spectrum of compound truxene in CDCl_3_. Figure S31: ^13^C NMR (101 MHz) spectrum of compound truxene in CDCl_3_. Figure S32: ^1^H NMR (400 MHz, DMSO-d6) spectrum for the oxidation of thioanisole catalyzed by Tru-COFs at 4 h (after the 1st cycle of the reaction). Figure S33: ^1^H NMR (400 MHz, DMSO-d6) spectrum for the oxidation of thioanisole catalyzed by Tru-COFs at 4 h (after the 3rd cycle of the reaction). Figure S34: ^1^H NMR (400 MHz, DMSO-d6) spectrum for the oxidation of methyl p-tolyl sulfide catalyzed by Tru-COFs at 4 h. Figure S35: ^1^H NMR (400 MHz, DMSO-d6) spectrum for the oxidation of 1-methoxy-4-(methylthio)benzene catalyzed by Tru-COFs at 4 h. Figure S36: ^1^H NMR (400 MHz, DMSO-d6) spectrum for the oxidation of 4-chlorothioanisole catalyzed by Tru-COFs at 4 h. Figure S37: ^1^H NMR (400 MHz, DMSO-d6) spectrum for the oxidation of 4-bromothioanisole catalyzed by Tru-COFs at 4 h. Figure S38: ^1^H NMR (400 MHz, DMSO-d6) spectrum for the oxidation of ethyl phenyl sulfide catalyzed by Tru-COFs at 4 h. Table S4: comparison of the reaction conditions and performances of different catalysts for photocatalytic selective oxidation of thioanisole. (a) Conversion (Conv.) and selectivity (Select.) were determined by ^1^H NMR. (b) The yield of benzyl methyl sulfoxide. (c) N.D. = not detected. Table S5: comparison of the reaction conditions and performances of different catalysts for photocatalytic sacrificial oxygen evolution. (a) Turnover number (TON) = the total number of moles of oxygen per mole of precatalyst. The amount of Tru-COFs was calculated based on the smallest repeating unit of the structure. (b) (O_2_ evolution) = (the amount of O_2_)/[(the mass of the catalyst) × (chemical reaction time)].
