# Supplementary material for: Synthesis of Two-Dimensional C–C Bonded Truxene-Based Covalent Organic Frameworks by Irreversible Brønsted Acid-Catalyzed Aldol Cyclotrimerization
Source: Research (Wash D C). 2021 Sep 3;2021:9790705. doi: 10.34133/2021/9790705 (PMC8435030; doi:10.34133/2021/9790705)
Supplement: Supplementary Materials — Scheme S1: synthesis of TDB. Scheme S2: synthesis of model compound truxene. Table S1: optimization of reaction conditions for Brønsted acid-catalyzed aldol cyclomerization of TDB. Figure S1: FT-IR spectra of TDB (red), the model compound truxene (green), and Tru-COFs (black). Figure S2: comparison of the solid-state 13C NMR (101 MHz) spectrum of Tru-COFs (top) with the model compound truxene (middle) and TDB (bottom). Figure S3: (a) top and (b) side view of the simulated structure of AA stacking and (c) top and (d) side view of the simulated structure of AB stacking. Table S2: unit cell parameters and fractional atomic coordinates for Tru-COF-based AA topology after unit cell correction and Pawley refinement. Figure S4: (a) 77 K N2 adsorption and desorption isotherms and (b) pore size distributions of Tru-COF sample synthesized with 0.2 equiv. PTSA. (c) 77 K N2 adsorption and desorption isotherms and (d) pore size distributions of Tru-COF sample synthesized with 0.4 equiv. PTSA. (e) 77 K N2 adsorption and desorption isotherms and (f) pore size distributions of Tru-COF sample synthesized with 0.6 equiv. PTSA. (g) 77 K N2 adsorption and desorption isotherms and (h) pore size distributions of Tru-COF sample synthesized with 0.8 equiv. PTSA. Figure S5: TGA profiles of Tru-COFs. Figure S6: WAXS profiles of Tru-COFs (blue) and recycled Tru-COFs after immersion in 9 M NaOH (black) and 9 M HCl (red) solutions for 24 h, respectively. Figure S7: FT-IR spectra of Tru-COFs (blue) and recycled Tru-COFs after immersion in 9 M NaOH (black) and 9 M HCl (red) solutions for 24 h, respectively. Figure S8: (a) 77 K N2 adsorption and desorption isotherms and (b) pore size distributions of Tru-COFs (blue) and recycled Tru-COFs after immersion in 9 M NaOH (black) and 9 M HCl (red) solutions for 24 h, respectively. Figure S9: the CV curves of the Tru-COFs measured in acetonitrile at 298 K. Figure S10: (a) solid ESR spectrum of Tru-COF sample, the ESR peak intensity was changed after Xe la [file 9790705.f1.doc]

**Supporting Information**

Synthesis of C–C Bonded Two-dimensional Truxene-based Covalent Organic Frameworks by Irreversible Brønsted Acid-catalyzed Aldol Cyclotrimerization

Qingsong Zhang^1,2^, Yunlong Sun^1,2^, Haijing Li^2,3^, Kun Tang^1,2^, Yu-Wu Zhong^1,2^, Dong Wang^1,2^, Yunlong Guo^1,2*^, and Yunqi Liu^1,2*^

^1^ Beijing National Laboratory for Molecular Sciences, Institute of Chemistry, Chinese Academy of Sciences, Beijing 100190, P. R. China

^2^ University of Chinese Academy of Sciences, Beijing 100049, P. R. China

3 Beijing Synchrotron Radiation Facility, Institute of High Energy Physics, Chinese Academy of Sciences, Beijing 100049, P. R. China.

**Content**

**General Methods**------------------------------------------------------S2

**Synthesis Procedure**--------------------------------------------------S4

**Rection condition optimization**------------------------------------S6

**Structure simulation**-------------------------------------------------S9

**Figures**------------------------------------------------------------------S12

**References**--------------------------------------------------------------S34

**General Methods**

The monomer TDB and model compound truxene were synthesized by Schleck technique in glassware under a dry argon atmosphere. The solvents we used were purified and dried by standard methods. The reagents that can be purchased commercially were used without further purification. Liquid NMR were recorded on a Bruker AVANCE 400. In detail, a 400 MHz NMR instrument was used for record ^1^H NMR spectra. The chemical shifts are reported in units of tetramethysilane and ppm, and the solvent resonance is the internal standard (CDCl_3_: *δ* 7.26 ppm). The data are reported as follows: chemical shift, multiplicity (s = singlet, d = doublet, t = triplet, q = quartet, br = broad, m = multiplet or unresolved), coupling constants (Hz), and integration. An NMR instrument operated at 101 MHz was used for record ^13^C NMR spectra with complete proton decoupling. Thin layer chromatography was used to monitor the reaction and visualized with UV light at 254 nm. The monomer TDB was purified by flash column chromatography with silica gel.

Using a standard CP pulse sequence probe with 4 mm (outside diameter) zirconia rotors, high resolution solid-state NMR spectra were recorded at ambient temperature on a Bruker AVANCE 400M Spectrometer. A Bruker Tensor-27 was used for record infrared spectra. Thermo gravimetric analysis (TGA) from 60~800 ℃ was carried out on a PE TGA8000 in nitrogen. A Bruker Autoflex-III mass spectrometer was used for record MALDI-TOF mass spectrometry in positive ion. A UV spectrophotometer (SHIMADZU, UV-2600) was used to record the solid ultraviolet visible light (UV/Vis) absorbance. An Auto Lab PGSTA 302 was used to record cyclic voltammetry (CV) experiment. The CV experiments were carried out in a three electrode electrochemical cell equipped with a salt bridge and a scan rate of 0.05 V/s, and conducted in dry acetonitrile. 0.1 M tetrabutylammonium hexafluorophosphate was used as electrolyte. Platinum flake-let was used as the auxiliary electrode. The Ag/Ag^+^ couple was used as the reference electrode. The working electrode was a glassy carbon electrode. The redox potential of Ferrocene/ferrocenium (Fc/Fc^+^) was measured at the end of each experiment in order to calibrate the pseudo reference electrode. COFs samples were milled and dispersed in ethanol with PTFE and stirred for 3 h (m_COF_/m_PTFE_ = 4/1). The suspension was then dropped on the glassy carbon electrode and dried to form thin films for measurements. Wide Angle X-Ray Scattering (WAXS) patterns were obtained on a Xenocs Xeuss 2.0 with Cu Kα line from 2θ = 3^o^ up to 37.5^o^. N_2_ adsorption isotherms were measured up to 1 bar at 77 K using an AUTOSORB-IQ-XR-C surface area analyzer. Prior to measurements, samples (50 mg) were degassed for over 12 h at 120 ℃. During the measurement, oil-free vacuum pumps and oil-free pressure regulators were used to avoid contamination of the samples. ESR experiments were tested on a Bruker E500 at room temperature. Materials Studio 8.0 suite of programs (Accelry Inc.). were used for molecular modeling of COF. Scanning eletron microscope (SEM) images were recorded on a Hitachi 4800 cold field scanning electron microscopy and a Zeiss Gemini 300 scanning electron microscopy. Transmission electron microscope (TEM) images were recorded on a JEM-2100F.

**Synthesis Procedure**


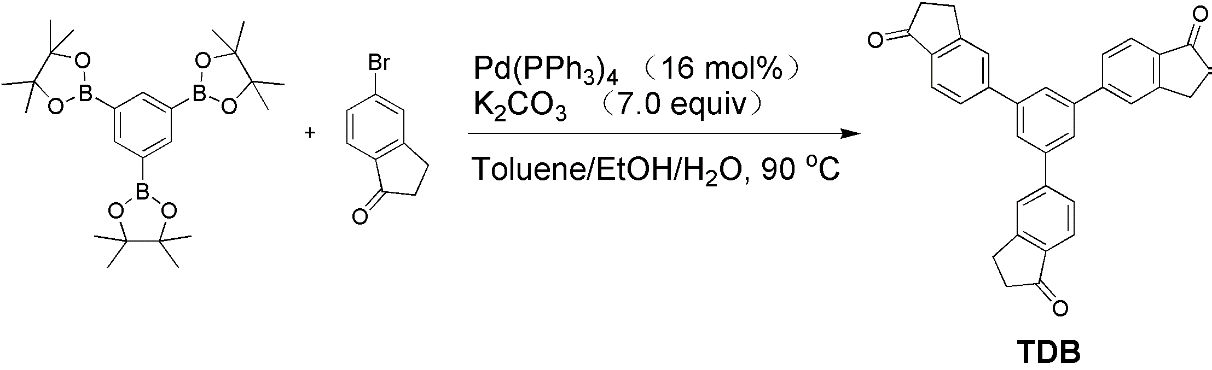


**Scheme S1**. Synthesis of **TDB**

**1,3,5-triindenonebenzene (TDB)**: The monomer TDB was synthesized through Suzuki-coupling, the method was previously reported, and we make a small change. [1] To a 500 mL three neck flask were added 5-bromo-indan-1-one (13.5 mmol, 4.5 equiv.), 1,3,5-tris(4,4,5,5-tetramethyl-1,3,2-dioxaborolan-2-yl) benzene (3 mmol, 1.0 equiv.), Pd(PPh_3_)_4_ (0.48 mmol, 0.16 equiv.), K_2_CO_3_ (21 mmol, 7 equiv.), 200 mL toluene, 20 mL H_2_O, and 20 mL ethanol. The mixture was stilled at 90 ^o^C, and kept for 24 h. Then the mixture was cooled to room temperature and filtrated to obtain a cake. The cake was washed with 20mL ethanol and 20 mL fresh toluene. The resulting cake was further purified by flash chromatography on silica gel, and using dichloromethane (DCM), ethyl acetate (EA) as the mixture eluent (DCM/EA = 5/1, R_f_ = 0.6). The product **TDB** (460 mg) was finally purified in 33% isolated yield. ^1^H NMR (400 MHz, CDCl_3_) *δ* 7.9~7.88 (m, 2H), 7.8 (s, 1H), 7.72 (d, J = 8 Hz, 1H), 3.26 (t, J = 12 Hz, 2H), 2.79 (t, J = 12 Hz, 2H); ^13^C NMR (101 MHz, CDCl_3_) *δ* 206.52, 156.06, 146.93, 142.04, 136.66, 127.07, 126.67, 125.55, 124.45, 36.66, 26.05; MALDI-TOF Calculated for C_33_H_24_O_3_: 468.17, found: 469.1795.


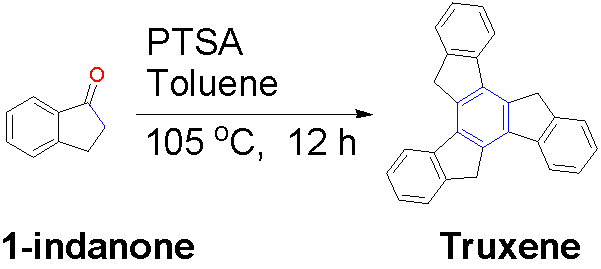


**Scheme S2.** Synthesis of model compound **truxene**

**T**ruxene: (truxene is a known compound, and can be purchased commercially). A Pyrex tube was filled with 1-indanone (26.4 mg, 0.2mmol, 1.0 equiv.), p-toluenesulfonic acid (PTSA) (38 mg, 0.2 mmol, 1.0 equiv.) and 1 mL dry toluene. The reaction mixture was degassed through three freeze-pump-thaw cycles, then sealed under vacuum and heated in an oven at 105 ^o^C for 24 h. The mixture was cooled down to room temperature, filtrated under vacuum. The resulting filter cake was washed with ethanol and acetone for several times, and dried under vacuum at 120 ^o^C for 4 h to afford the light-yellow powder (15 mg) in 66% isolated yield. ^1^H NMR (400 MHz, CDCl3) *δ* 7.97 (d, J = 8 Hz, 3H), 7.71 (d, J = 8 Hz, 3H), 7.51 (t, J = 16 Hz, 3H), 7.40 (t, J = 16 Hz, 3H), 4.29 (s, 6H). ^13^C NMR (101 MHz, CDCl3) *δ* 143.98, 141.91, 137.33, 135.47, 127.11, 126.48, 125.30, 122.07, 36.74. HRMS (EI) Calculated for C_27_H_18_: 342.1409, found: 342.1399.

**Reaction condition optimization**

In order to get crystallized product, we have screened the reaction conditions by changing the solvent, temperature and catalyst. After many trials, we finally find the right condition to obtain Tru-COFs in moderate crystallinity.

Table S1. Optimization of reaction conditions for Brønsted acid-catalyzed Aldol cyclomerization of TDB. *^a^* ND = not determined. *^b^* [BMIM][BF4] = 1-butyl-3-methylimidazolium tetrafluoroborate. *^c^* MC = moderate crystallinity. All reactions are carried out in a Pyrex tube without stirred and placed in an oven at a set temperature for three days.

| **Entry** | **Solvent** | **T**  **(**℃**)** | **PTSA**  (equiv.) | **Product** | **BET** **surface area**  (m^2^/g) | **Yield**  (%) |
| --- | --- | --- | --- | --- | --- | --- |
| 1 | 1,4-dioxane | 105 | 1.0 | No Solid | *a* | *a* |
| 2 | o-dichlorobenzene | 105 | 1.0 | Amorphous | *a* | 80.2 |
| 3 | [BMIM][BF4]*^b^* | 105 | 1.0 | No Solid | *a* | *a* |
| 4 | Toluene | 90 | 1.0 | Amorphous | *a* | *a* |
| 5 | Toluene | 105 | 0.2 | Amorphous | 45 | *a* |
| 6 | Toluene | 105 | 0.4 | Amorphous | 107 | *a* |
| 7 | Toluene | 105 | 0.6 | MC*^c^* | 657.8 | 38.7 |
| 8  9 | Toluene  Toluene | 105  105 | 0.8  1.0 | MC  MC | 643.2  645 | 55.6  74.8 |

**Photocatalytic test**

Photocatalytic oxidation of sulfide was measure in a 100 mL glass bottle. The reaction was carried out by using 0.25 mmol of sulfide, 5 mg (0.036 mmol) of Tru-COFs (the amount is based on the smallest repeating unit of the structure in the polymer), 5 mL CH_3_CN, 300 W Xe lamp (λ > 420 nm) and 1 atm air at 25 ^o^C.

Photocatalytic O_2_ production was measured in a Pyrex top-irradiation reaction vessel. The top of the vessel was connected to a glass closed gas circulation system. Catalyst powder (5.0 mg Tru-COFs) was dispersed in an aqueous solution (100 mL) containing Na_2_S_2_O_8_ (0.01 M) and 10 mM buffer solution (NaH_2_PO_4_/Na_2_HPO_4_ = 0.7/4.3, pH = 8). The solution was evacuated for several times to remove the dissolved oxygen. The reaction vessel was cooled by water and irradiated under a 300 W Xe lamp (λ > 360 nm). A gas chromatography equipped with a thermal conductive detector was used to analyze the evolved gases.


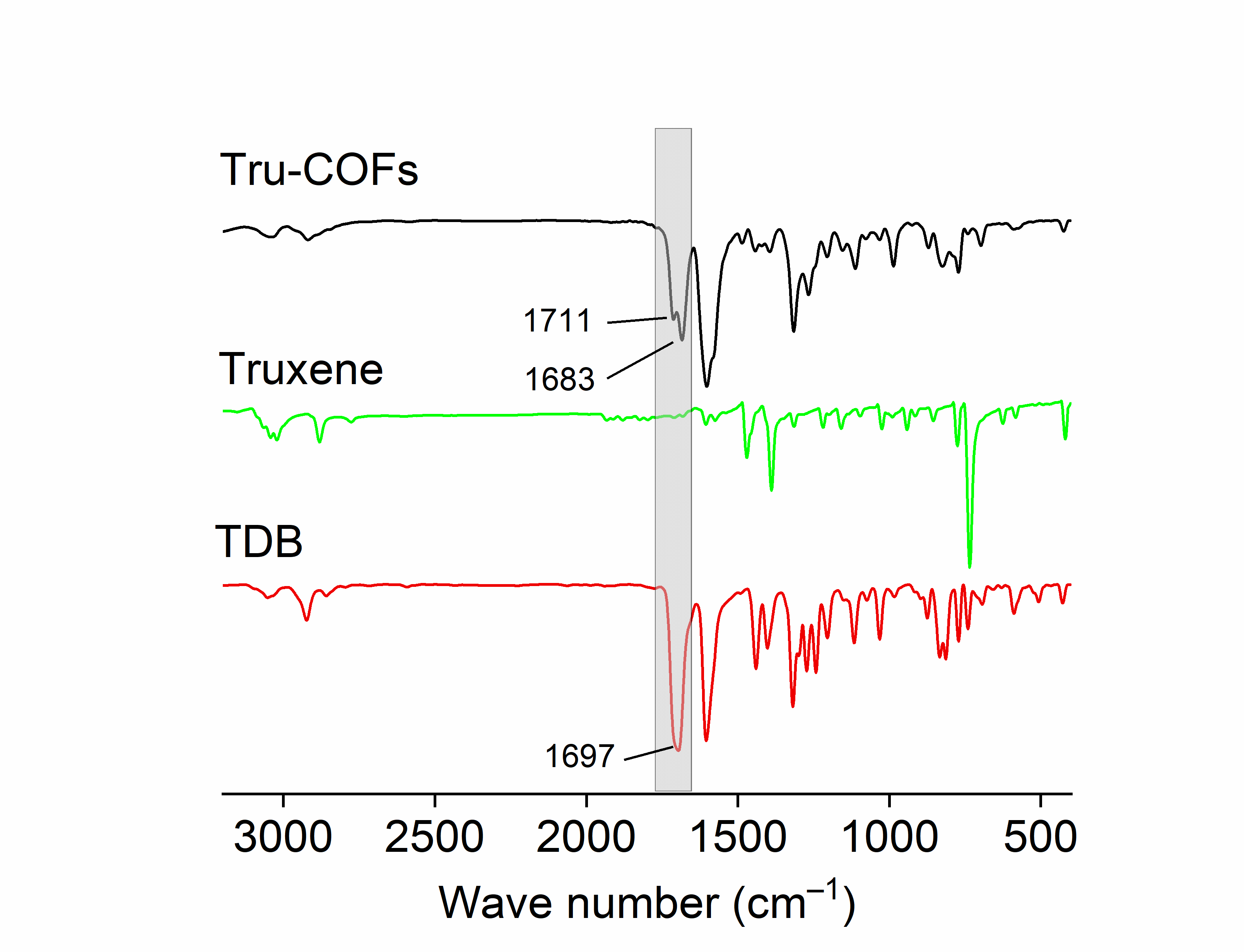


**Figure S1.** FT-IR spectra of TDB (red), the model compound Truxene （green） and Tru-COFs (black).


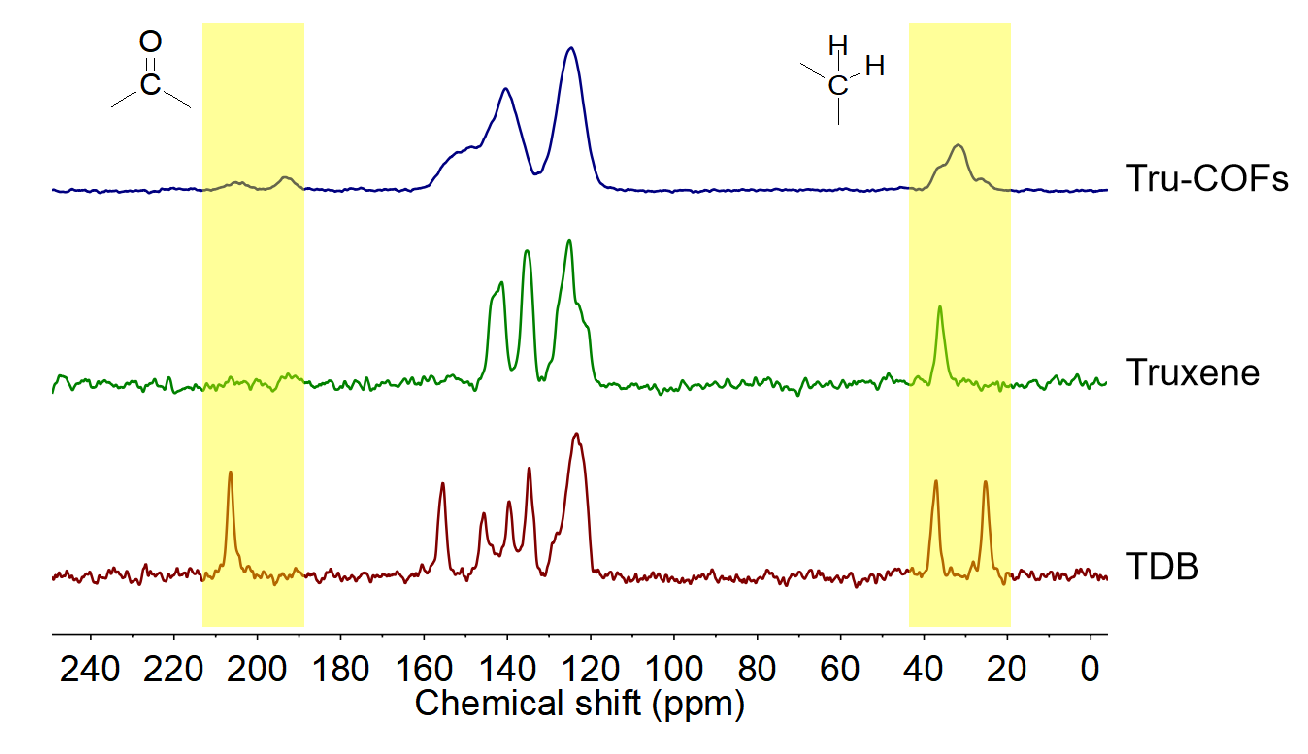


**Figure S2**. Comparison of the Solid State ^13^C CP/MAS NMR (101 MHz) spectrum of Tru-COFs (top) with the model compound Truxene (middle) and TDB (bottom).

**Structure Simulation**


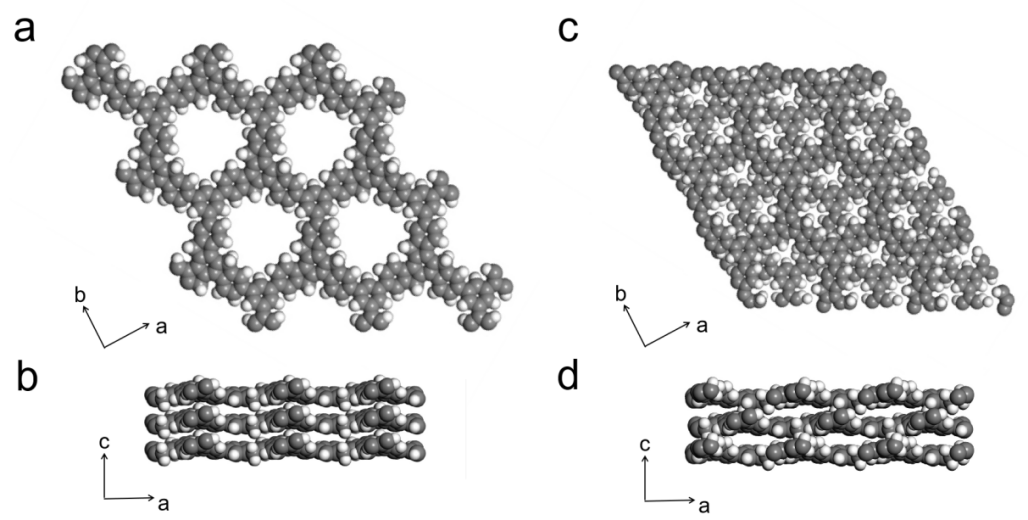


**Figure S3.** (a) Top and (b) side view of the simulated structure of AA stacking and (c) Top and (d) side view of the simulated structure of AB stacking. Color code: H white, C gray.

**Table S2.** Unit cell parameters and fractional atomic coordinates for Tru-COFs based AA topology after unit cell correction and Pawley refinement

| Space group | P1 | | |
| --- | --- | --- | --- |
| Calculated unit cell | *a* = 14.4074 Å, *b* = 14.4061 Å, *c* = 3.4850 Å  *α* = 90^o^, *β* = 90^o^, *γ* = 120^o^ | | |
| Atom | X | Y | Z |
| C | 2.8986 | –1.51209 | –0.13432 |
| C | 2.9665 | –1.55336 | –0.21446 |
| C | 3.07661 | –1.48574 | –0.19973 |
| C | 3.11986 | –1.37857 | –0.12611 |
| C | 3.05582 | –1.3346 | –0.05854 |
| C | 2.94566 | –1.40151 | –0.05924 |
| C | 3.16008 | –1.51808 | –0.19632 |
| C | 3.25616 | –1.41532 | –0.08799 |
| C | 3.23253 | –1.33379 | –0.06279 |
| C | 3.35945 | –1.39379 | –0.00909 |
| C | 3.43732 | –1.29225 | 0.09868 |
| C | 3.4131 | –1.20988 | 0.1139 |
| C | 3.31164 | –1.23003 | 0.02528 |
| C | 3.48053 | –1.09541 | 0.16033 |
| C | 3.42072 | –1.04858 | 0.07933 |
| C | 3.30817 | –1.12897 | –0.00636 |
| C | 3.40713 | –1.46077 | –0.02279 |
| C | 3.51205 | –1.40024 | 0.08039 |
| C | 3.54081 | –1.28805 | 0.16938 |
| C | 3.58751 | –1.03381 | 0.25154 |
| C | 3.63457 | –0.92208 | 0.24591 |
| C | 3.5758 | –0.87276 | 0.13718 |
| C | 3.46679 | –0.93867 | 0.05636 |
| C | 3.3646 | –1.56625 | –0.1306 |
| C | 3.43057 | –1.61108 | –0.1279 |
| C | 3.53776 | –1.55073 | –0.01697 |
| C | 3.57854 | –1.44376 | 0.08833 |
| C | 3.60647 | –1.59944 | –0.0121 |
| C | 3.56557 | –1.7049 | 0.09811 |
| C | 3.6273 | –1.75474 | 0.08415 |
| C | 3.73606 | –1.69198 | –0.00012 |
| C | 3.78093 | –1.58329 | –0.09057 |
| C | 3.71286 | –1.54063 | –0.11683 |
| H | 2.93586 | –1.6367 | –0.272 |
| H | 3.0889 | –1.25221 | 0.01554 |
| H | 2.89836 | –1.36669 | 0.02113 |
| H | 3.17182 | –1.5424 | –0.45845 |
| H | 3.13983 | –1.58316 | –0.00598 |
| H | 3.28906 | –1.11762 | –0.27625 |
| H | 3.25185 | –1.12489 | 0.17871 |
| H | 3.60568 | –1.22913 | 0.00019 |
| H | 3.56301 | –1.27081 | 0.44779 |
| H | 3.63386 | –1.07028 | 0.32519 |
| H | 3.7169 | –0.87498 | 0.32343 |
| H | 3.4184 | –0.90669 | –0.03956 |
| H | 3.28341 | –1.6128 | –0.21938 |
| H | 3.39844 | –1.69235 | –0.21792 |
| H | 3.6598 | –1.39561 | 0.17738 |
| H | 3.48483 | –1.74801 | 0.19484 |
| H | 3.78546 | –1.72785 | 0.0053 |
| H | 3.74217 | –1.46102 | –0.21838 |


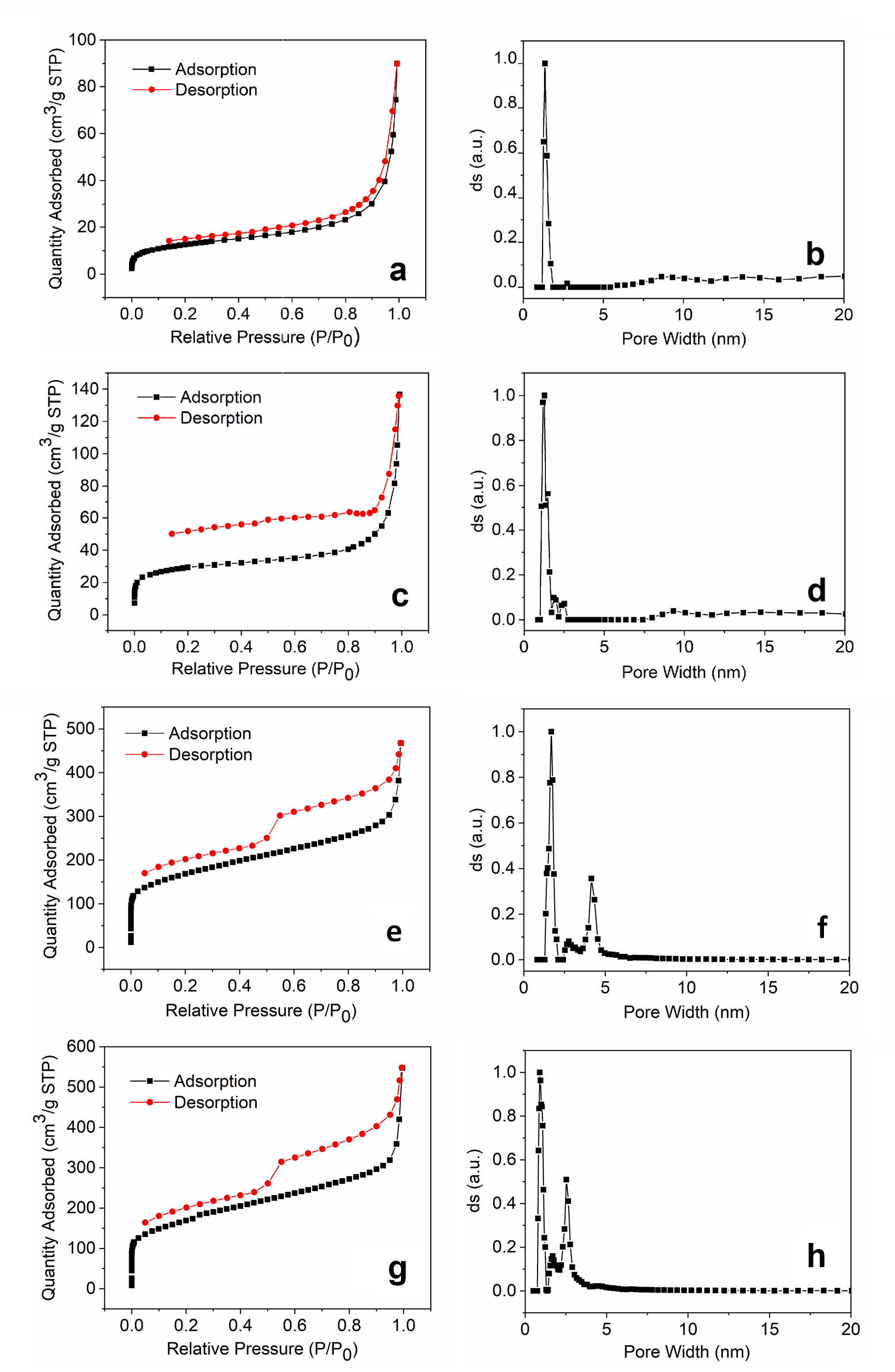


**Figure S4**. (a) 77 K N_2_ adsorption and desorption isotherms and (b) pore size distributions of Tru-COFs sample synthesized with 0.2 equiv. PTSA. (c) 77 K N_2_ adsorption and desorption isotherms and (d) pore size distributions of Tru-COFs sample synthesized with 0.4 equiv. PTSA. (e) 77 K N_2_ adsorption and desorption isotherms and (f) pore size distributions of Tru-COFs sample synthesized with 0.8 equiv. PTSA. (g) 77 K N_2_ adsorption and desorption isotherms and (h) pore size distributions of Tru-COFs sample synthesized with 1.0 equiv. PTSA.


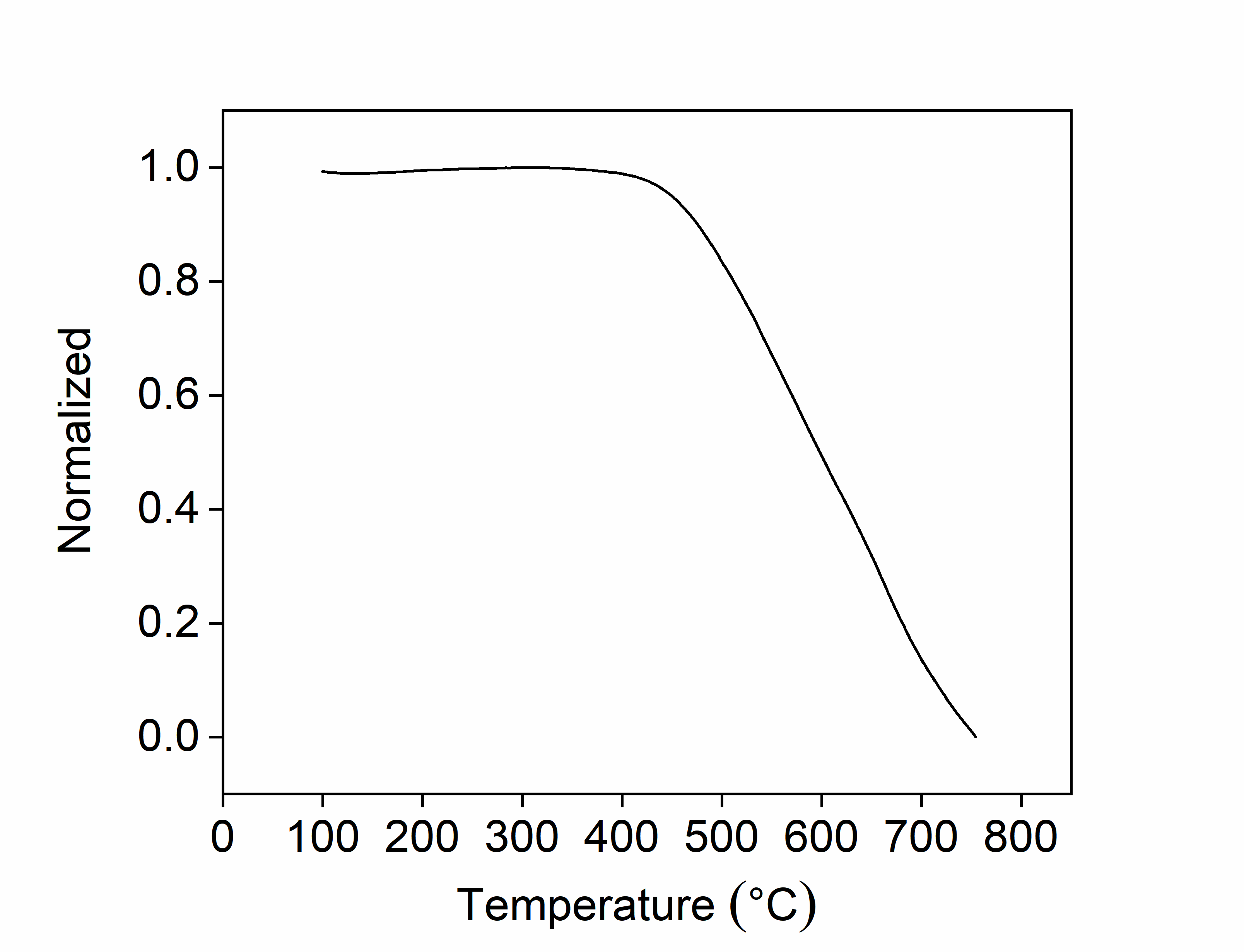


**Figure S5.** TGA profiles of Tru-COFs.


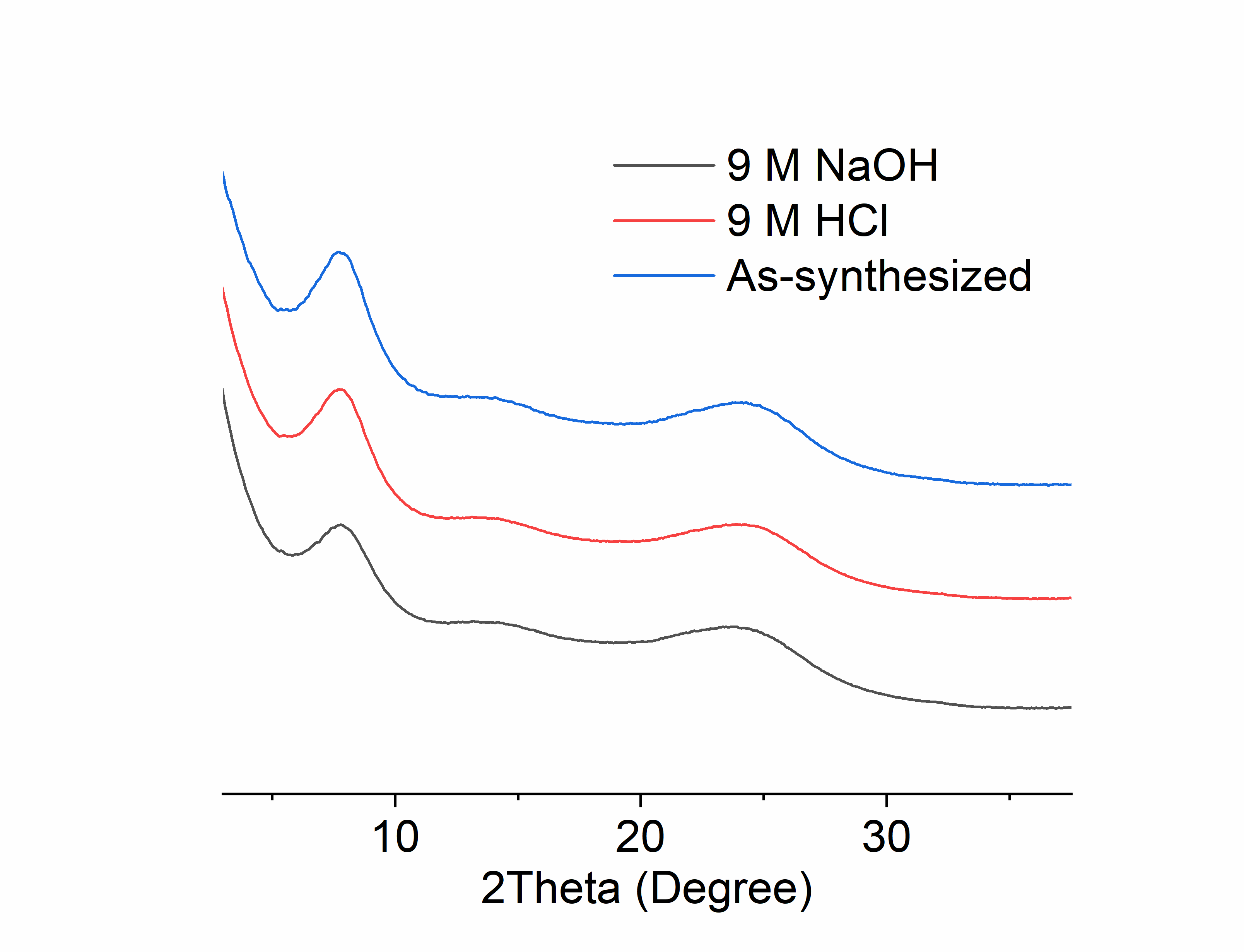


**Figure S6.** WAXS profiles of Tru-COFs (Blue) and recycled Tru-COFs after immersed in 9 M NaOH (black) and 9 M HCl (red) solutions for 24 h, respectively. Only small change was observed in 9 M NaOH, and no change was observed in 9 M HCl, which indicate the superior chemical stability of Tru-COFs**.**

**
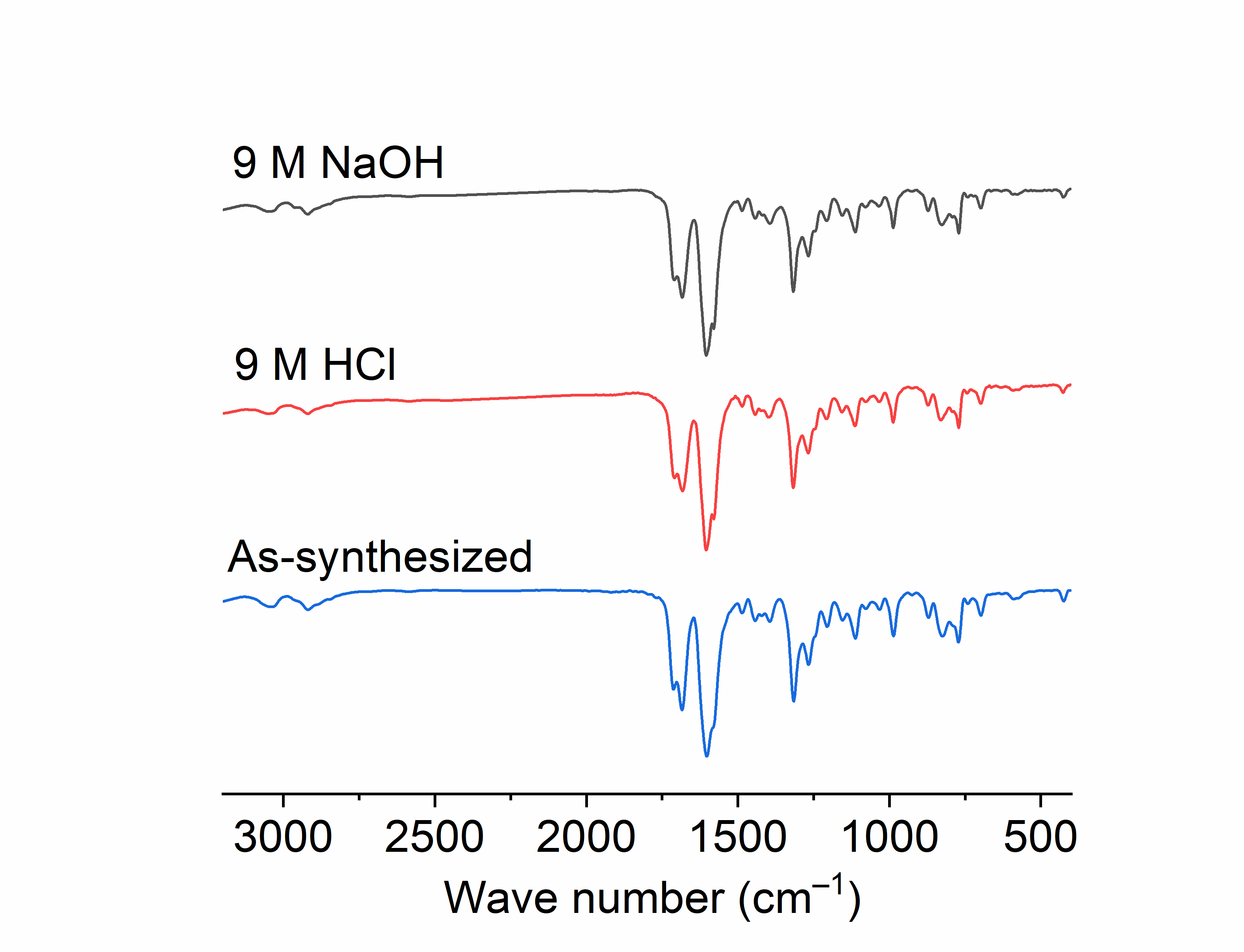
**

**Figure S7.** FT-IR spectra of Tru-COFs (blue) and recycled Tru-COFs after immersed in 9 M NaOH (black) and 9 M HCl (red) solutions for 24 h, respectively. The unchanged FT-IR spectra indicate the superior chemical stability of Tru-COFs**.**

**
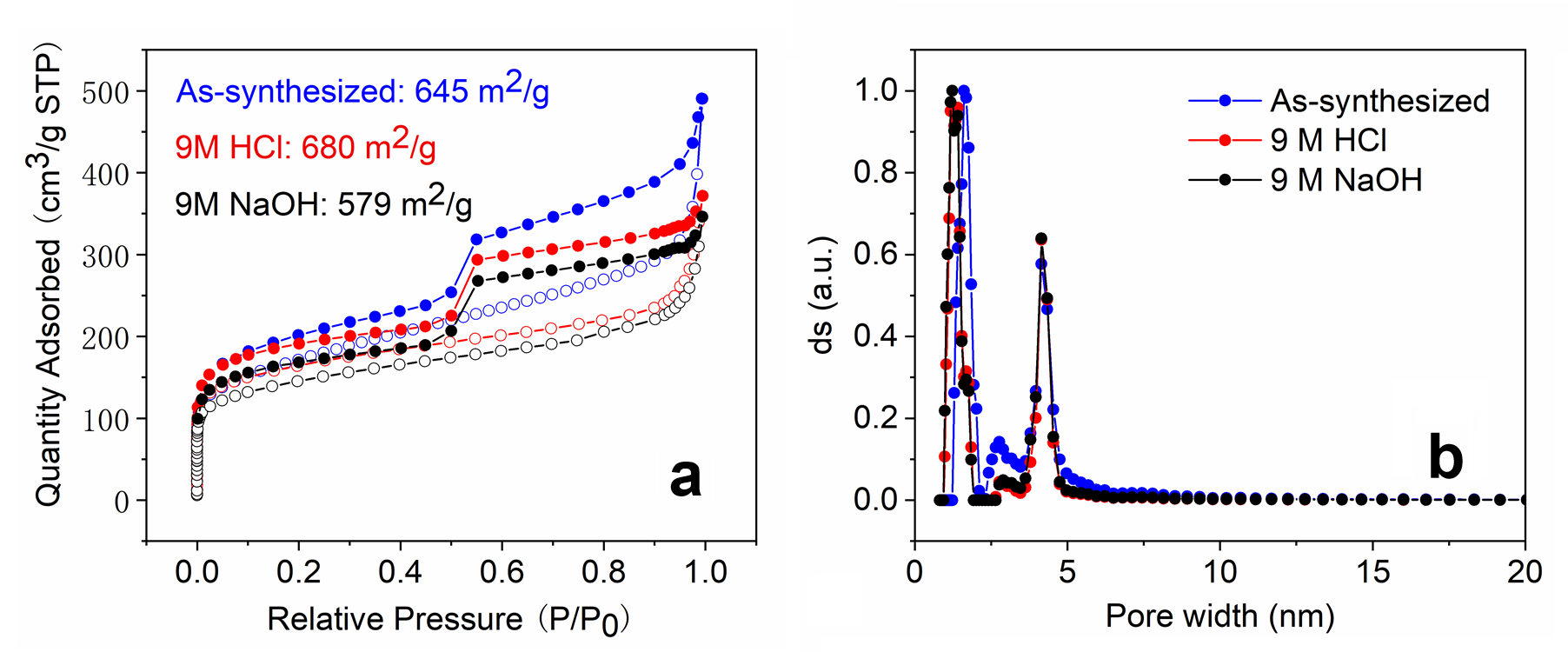
**

**Figure S8.** (a) 77 K N_2_ adsorption and desorption isotherms and (b) pore size distributions of Tru-COFs (blue) and recycled Tru-COFs after immersed in 9 M NaOH (black) and 9 M HCl (red) solutions for 24 h, respectively.


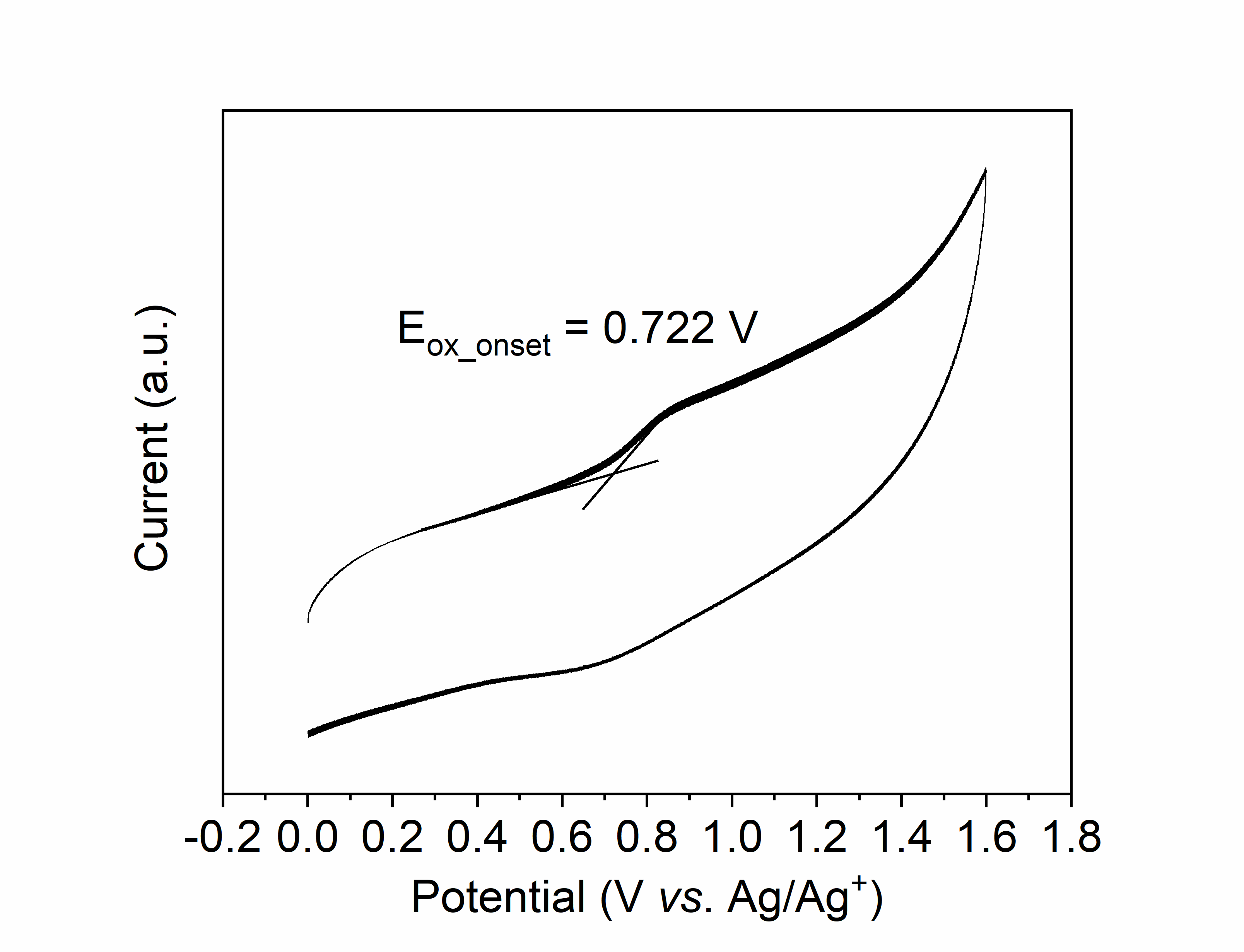


**Figure S9**. The CV curves of the Tru-COFs measured in acetonitrile at 298 K.


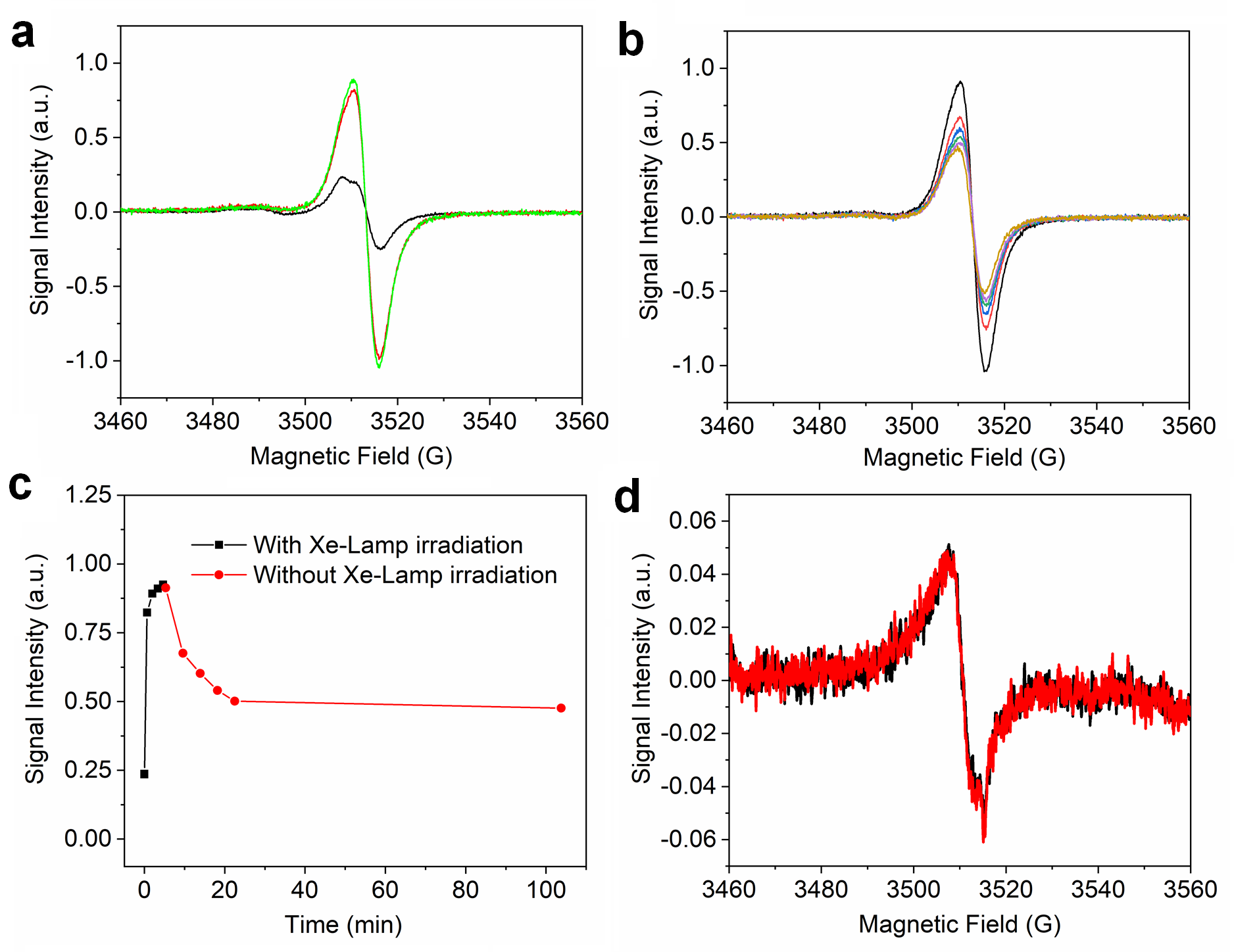


**Figure S10**. (a) Solid ESR spectrum of Tru-COFs sample, the ESR peak intensity was changed after Xe lamp irradiation, black line is the dark reaction, red line is the sample irradiated for 40 sec, green line is the sample irradiated for 120 sec. (b) Solid ESR spectrum of Tru-COFs sample, color black stands for the material photo initiated radicals up to saturation, the Xe lamp was then turned off, and the ESR experiment was done every 4.3 min once, the signal intensity was getting weaker obviously. (c) Plot of peak-to-peak height of the ESR signals versus irradiation time. The peak intensity was saturated after 120 sec and decreased after the Xe lamp was turned off. (d) Solid ESR spectrum of model compound truxene showed weak signal intensity in dark reaction for the absorbance of ambient light (black line), and the absorbance was already saturated for there was nothing changed with the Xe lamp irradiation (red line).


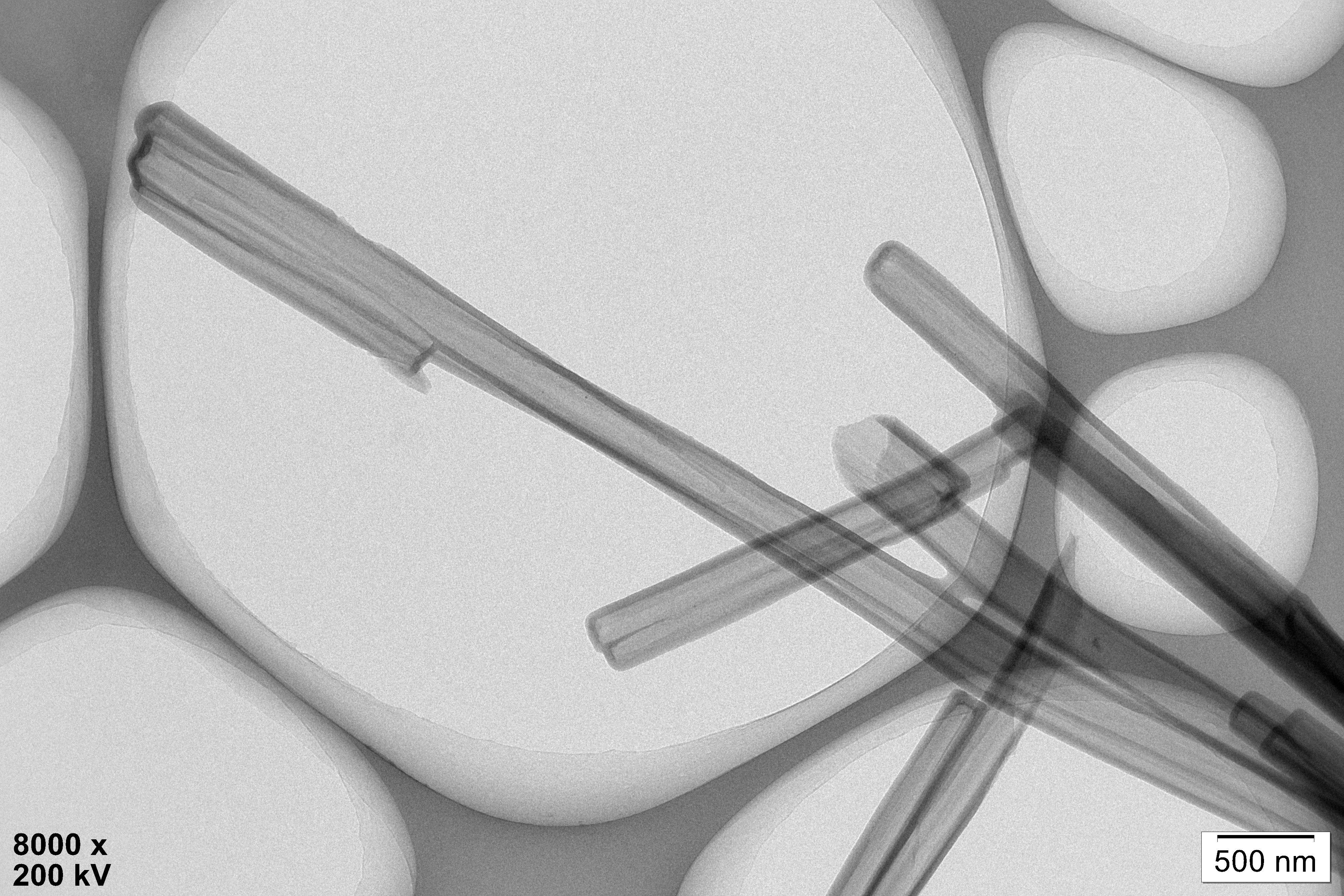


**Figure S11**. TEM image of Tru-COFs.


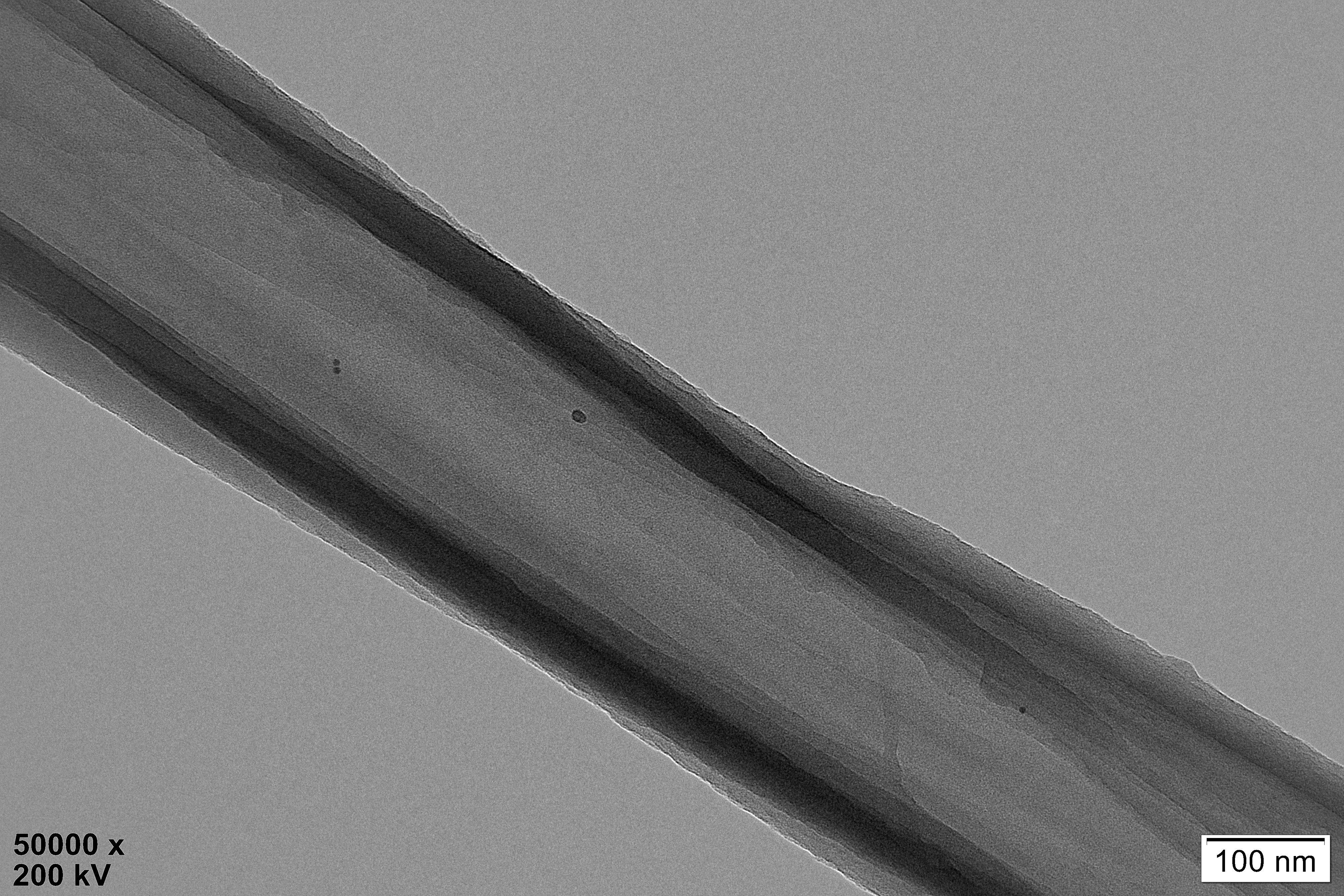


**F****igure S12**. TEM image of Tru-COFs.


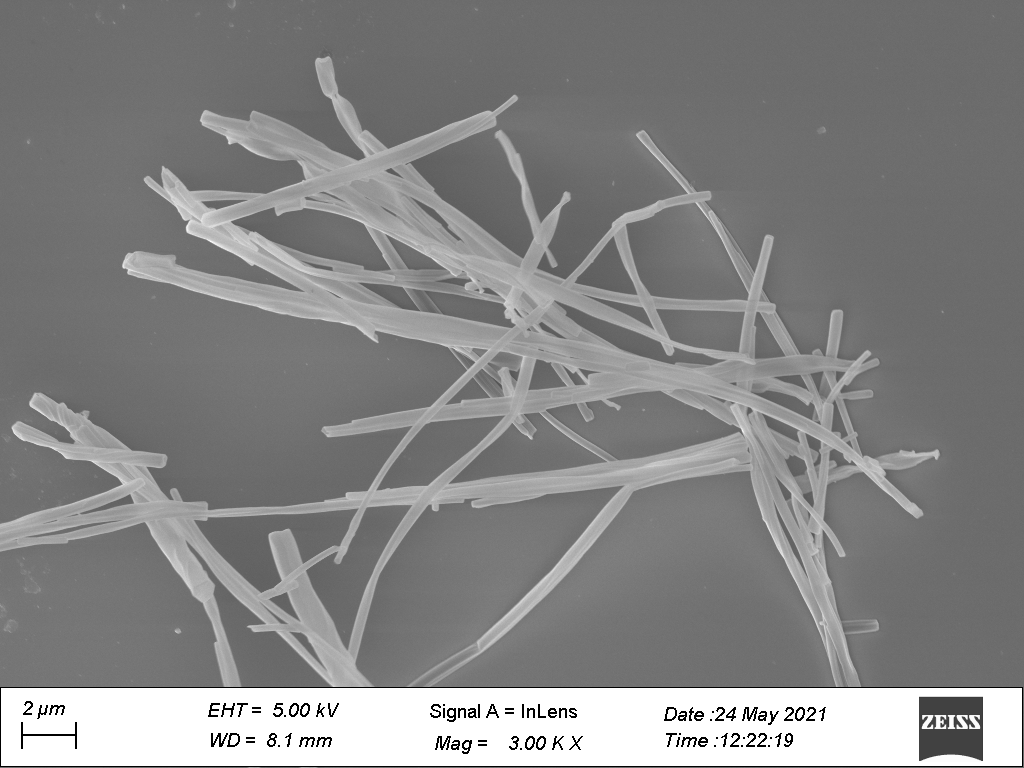


**Figure S13**. SEM image of Tru-COFs.

**Table S3.** Recycle test of Tru-COFs in thioanisole oxidation reaction. Conditions: air (1 atm), 0.25 mmol thioanisole, 5 mg (0.036 mmol) Tru-COFs, 5 mL CH_3_CN, 4h, 300 W Xe lamp, λ > 420 nm, 25 ^o^C. The conversion of the reaction and the selectivity of sulfoxide were determined by ^1^H NMR.


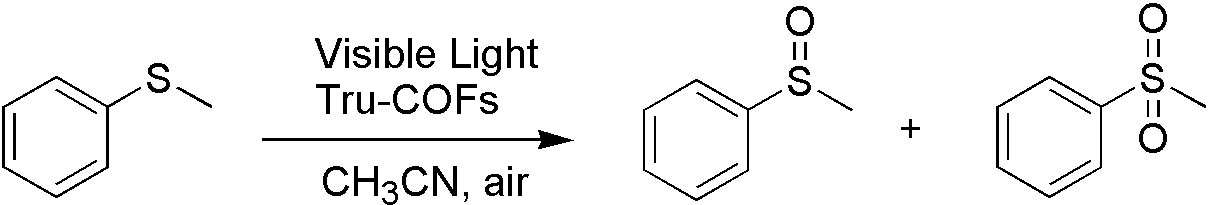


| **Entry** | **T (**h**)** | **Conv (**%**)** | **Select (**%**)** |
| --- | --- | --- | --- |
| 1 | 4 | 100 | 90 |
| 2 | 4 | 100 | 94 |
| 3 | 4 | 100 | 94 |


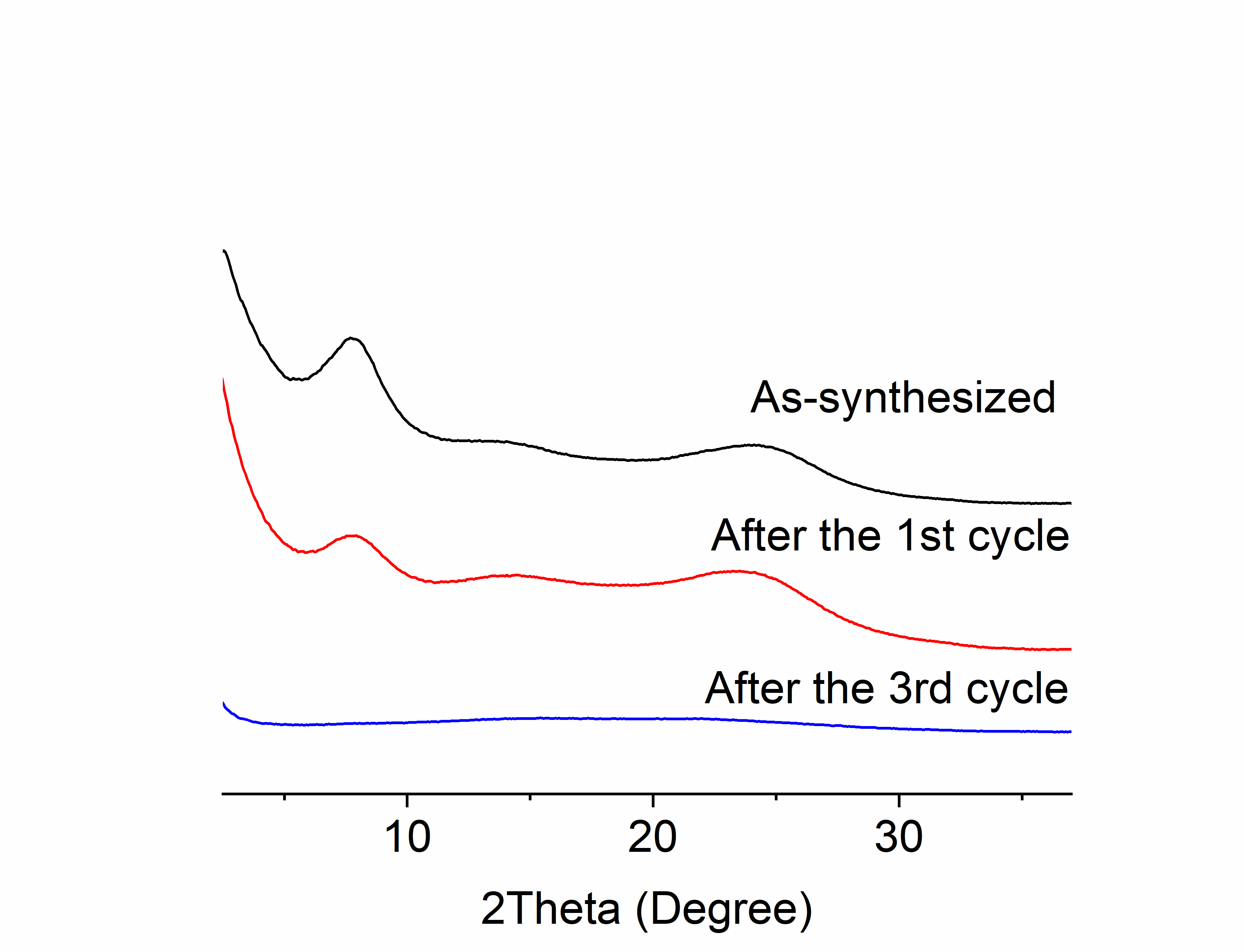


**Figure S14**. WAXS profile of Tru-COFs (black) and Tru-COFs after the 1st cycle (red) and Tru-COFs after the 3rd cycle (blue) of photocatalytic oxidation of thioanisole.


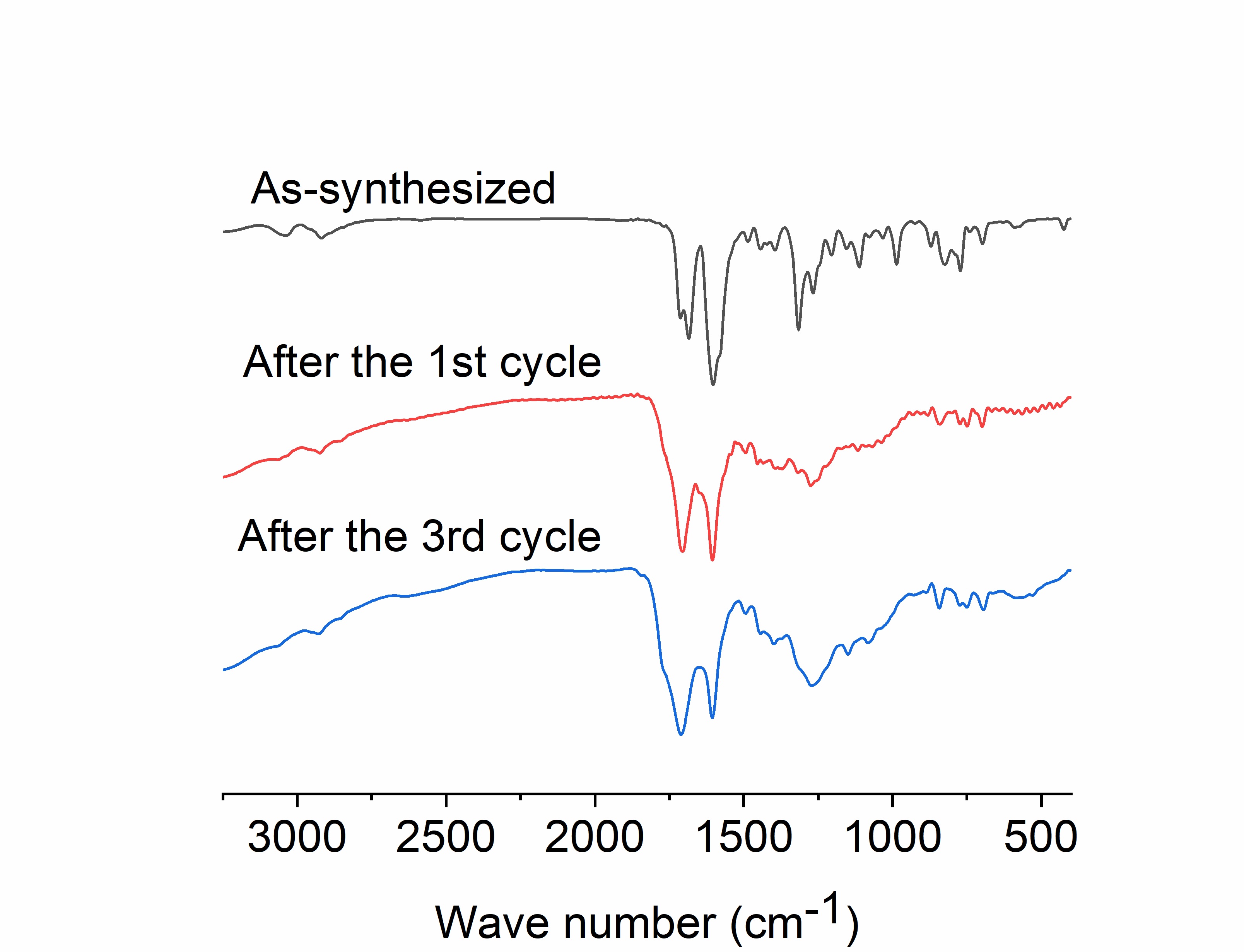


**Figure S15**. FT-IR spectra of Tru-COFs (black), Tru-COFs after the 1st cycle (red) and Tru-COFs after the 3rd cycle (blue) of photocatalytic oxidation of thioanisole.


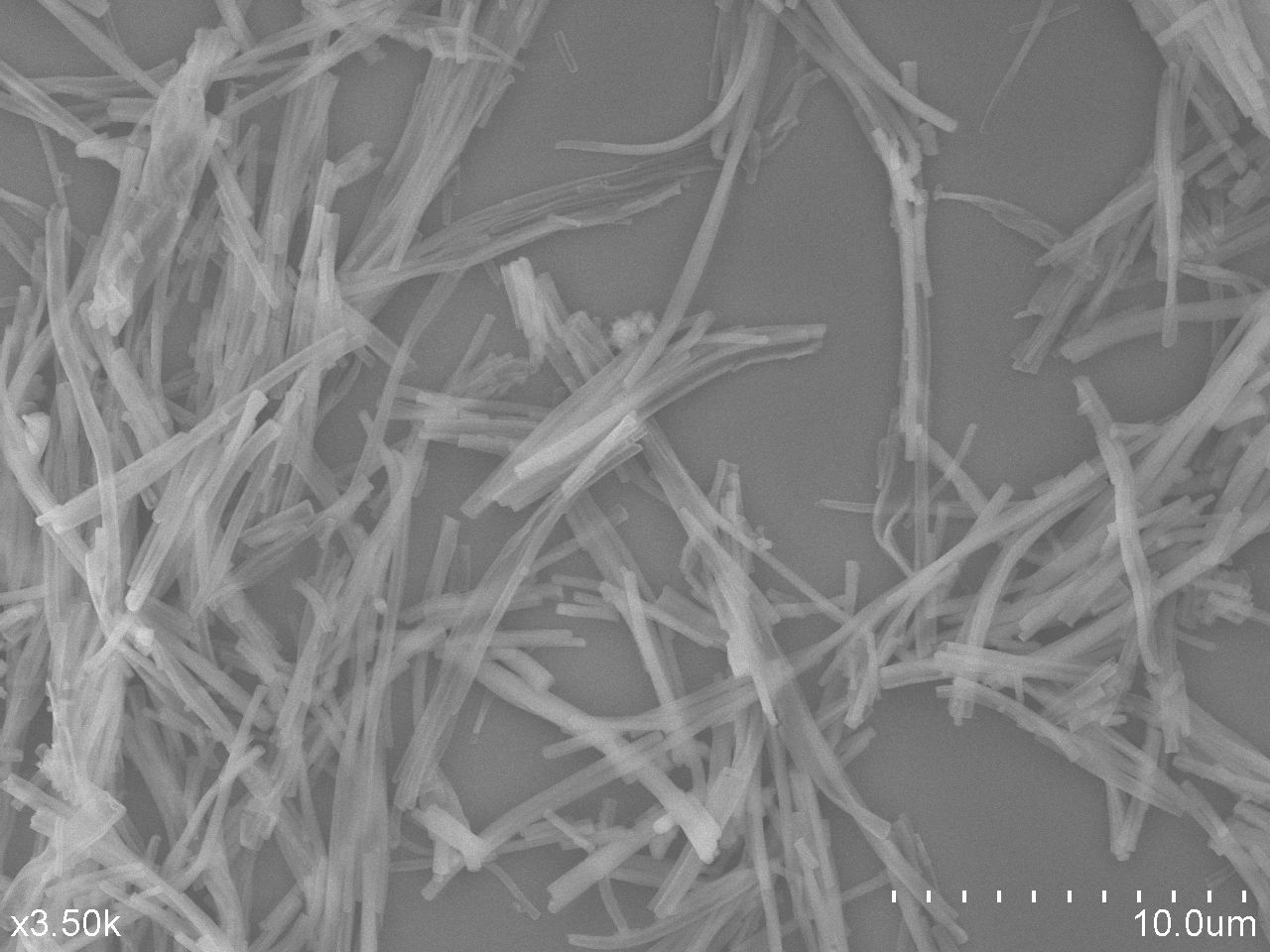


**Fig**ure **S16**. SEM image of Tru-COFs after the 1st cycle.


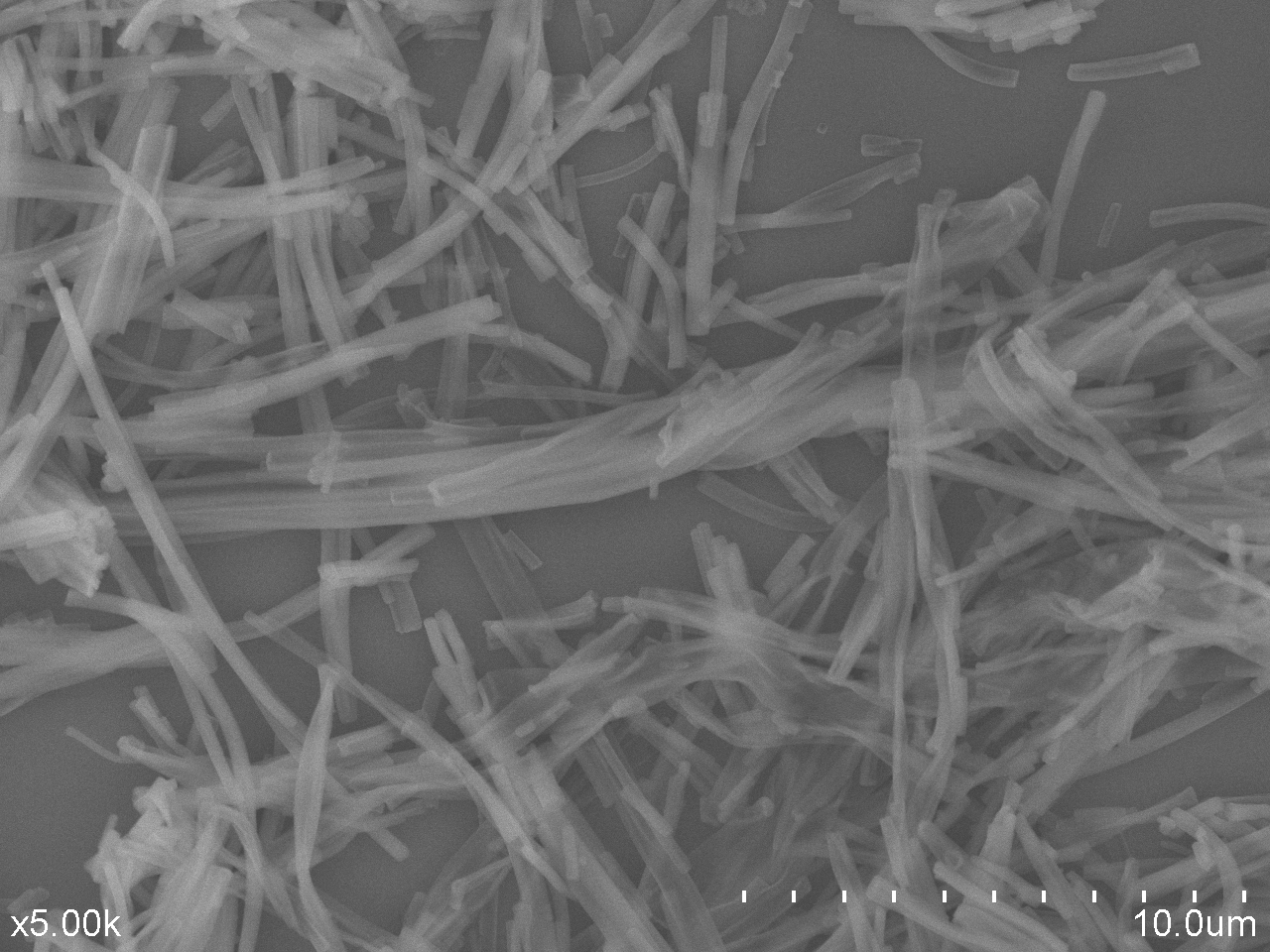


**Figure S17**. SEM image of Tru-COFs after the 3rd cycle.


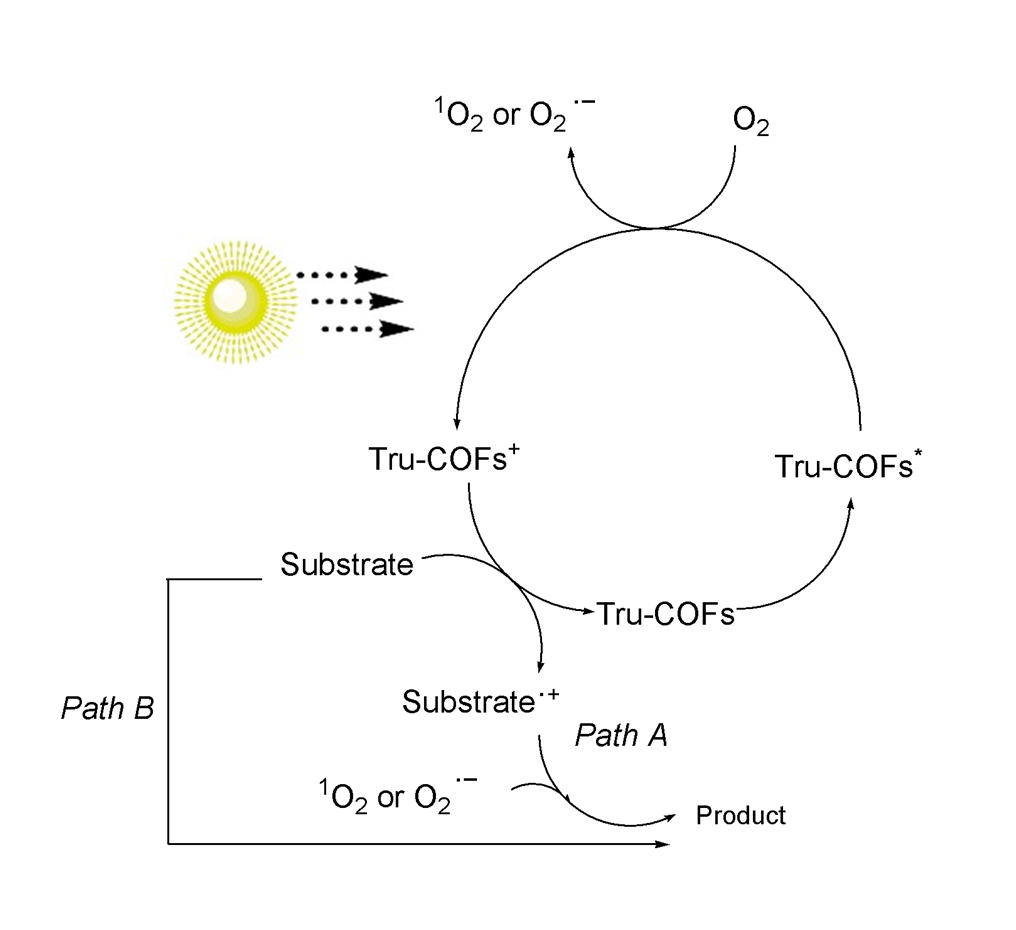


**Figure S18.** A plausible mechanism for Tru-COFs catalyzed the visible-light-driven selective oxidation of sulfides [6].


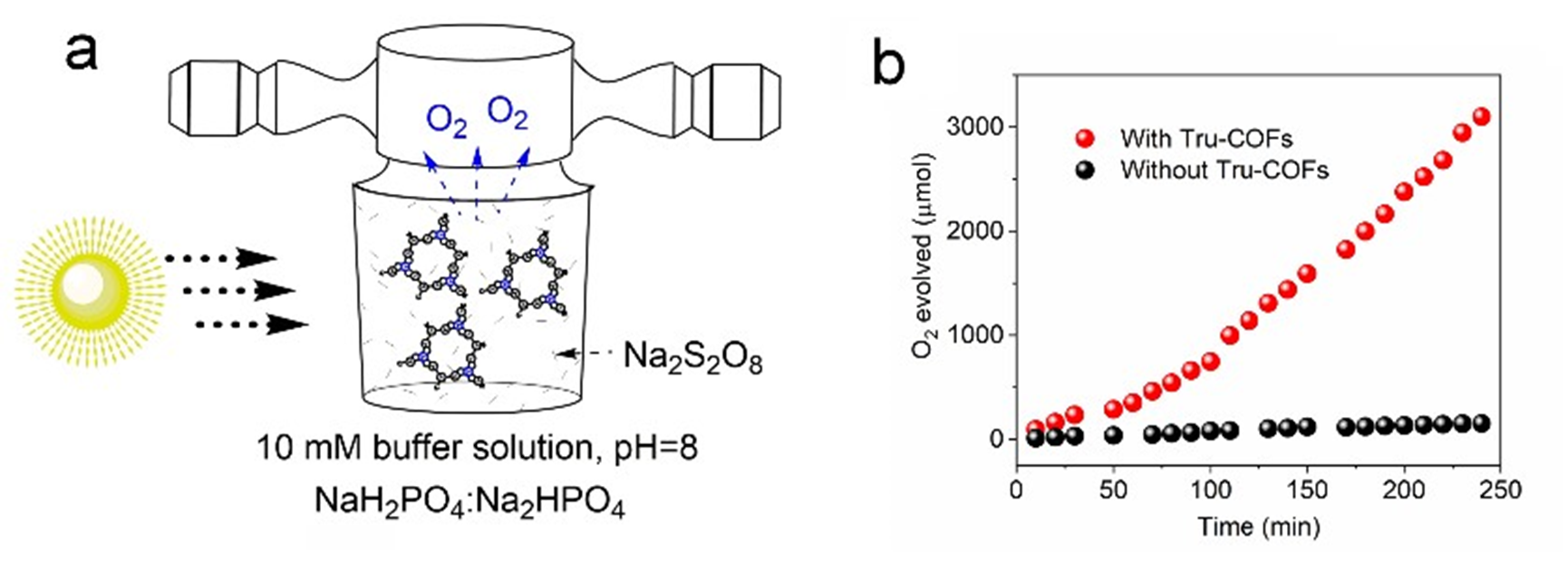


**Figure 19.** (a) Schematic of photocatalyzed sacrificial oxygen evolution. Using Na2S2O8 as sacrificial reagent in 10 mM buffer solution, n(Na_2_HPO_4_)/n(NaH_2_PO_4_) = 6.17, pH = 8. (b) Sacrificial oxygen evolution experiment with 5 mg Tru-COFs as WOC (red line) and without Tru-COFs (black line).

Tru-COFs showed a much better sacrificial oxygen evolution (150 μmol/gh) than the reaction without the Tru-COFs. The initial change in the slope in Figure 4c might be due to the gradual saturation of radical generation in Tru-COFs (Figure S10). The mechanistic step of sacrificial oxygen evolution possibly included light-activated e^–^/h^+^ pair generation and separation, followed by water oxidation and the reduction of the Na_2_S_2_O_8_ sacrificial agent (Figure 4).


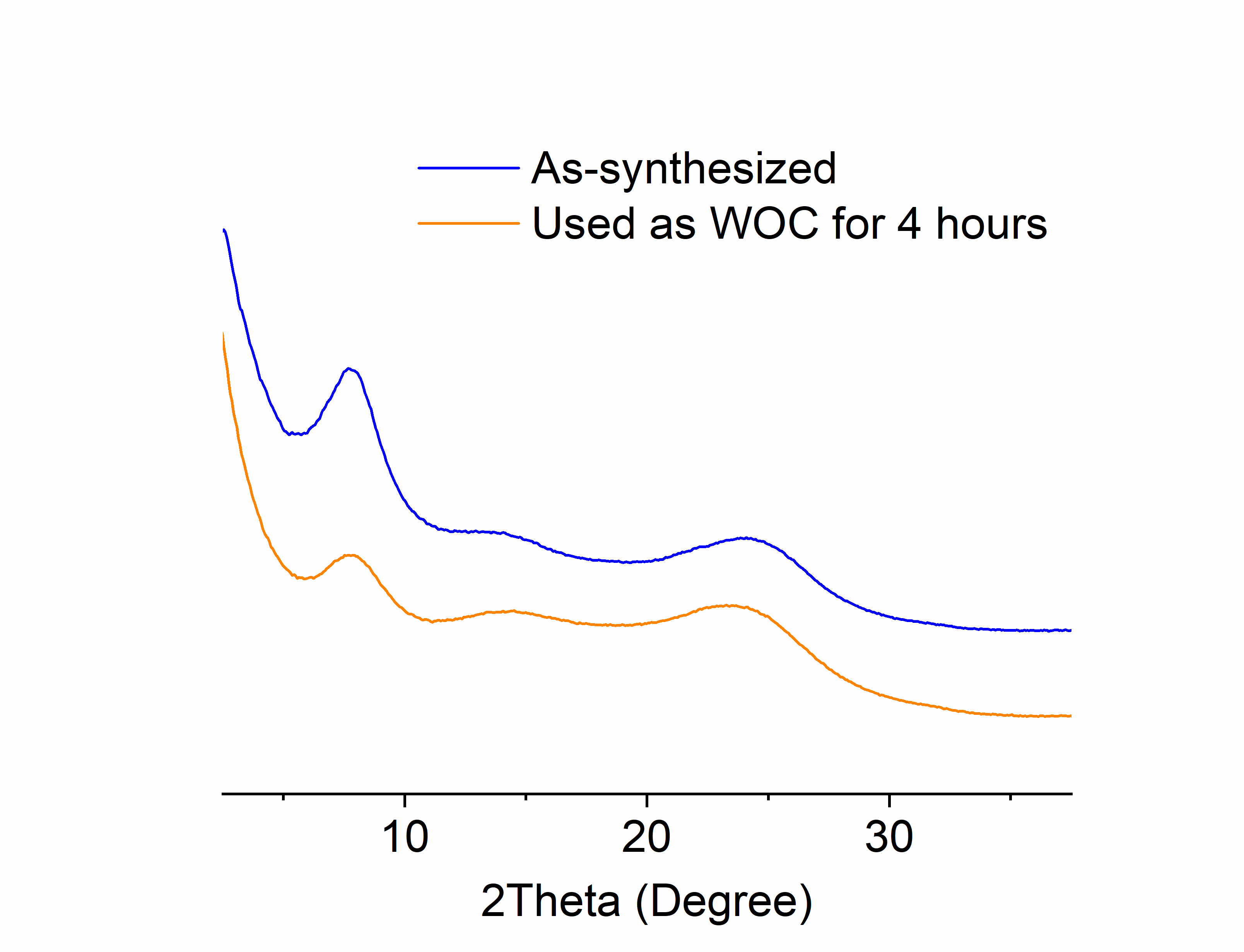


**Figure S20**. WAXS profile of Tru-COFs (blue) and recycled Tru-COFs (yellow) after used as water oxidation catalyst (WOC) for 4 h. Compared with the as-prepared sample, the WAXS of Tru-COFs (used for 4 h) showed a weaker signal of the (100) peak.


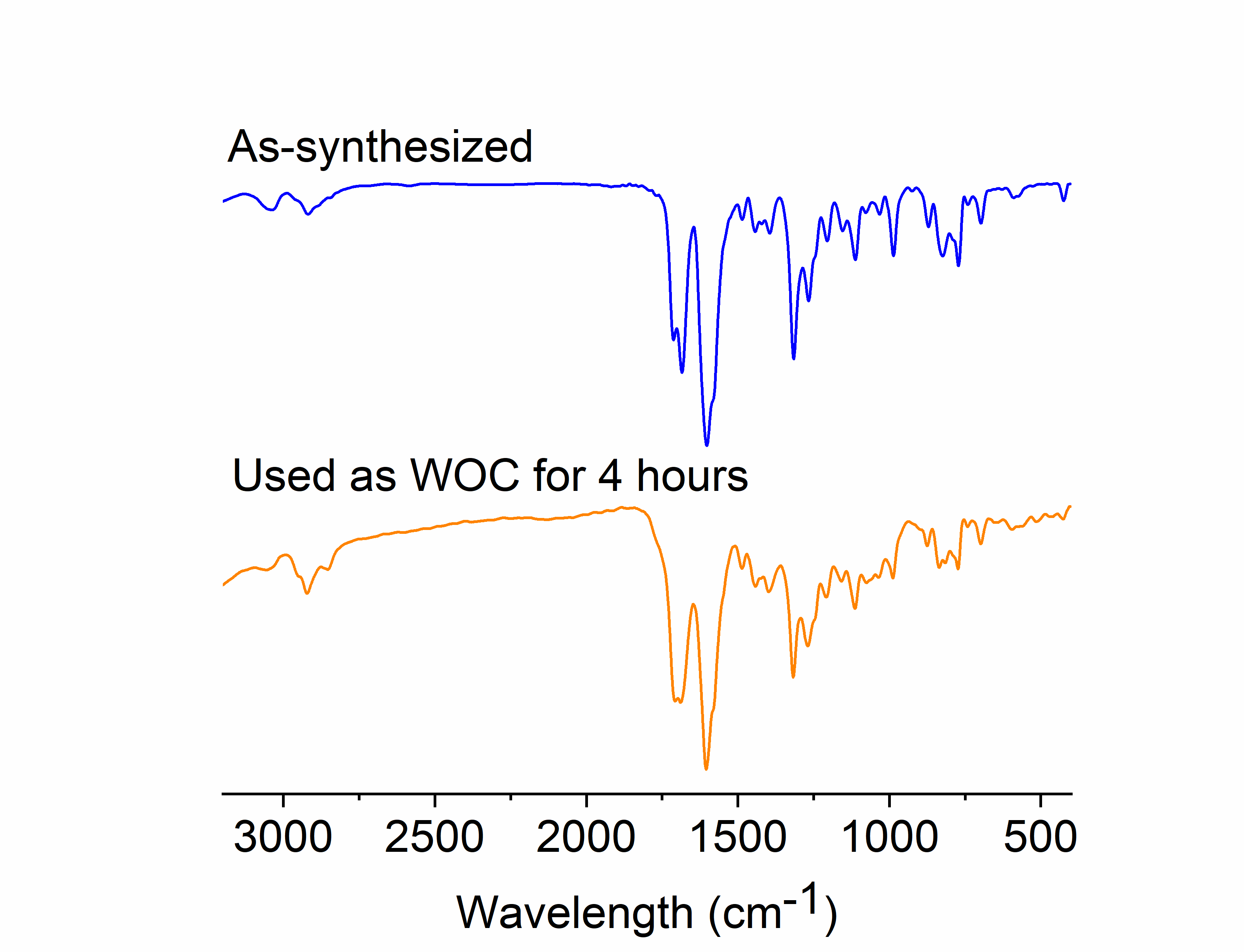


**Figure S21**. FT-IR of Tru-COFs (blue) and recycled Tru-COFs (yellow) after used as water oxidation catalyst (WOC) for 4 h. The FT-IR spectrum showed almost no change.


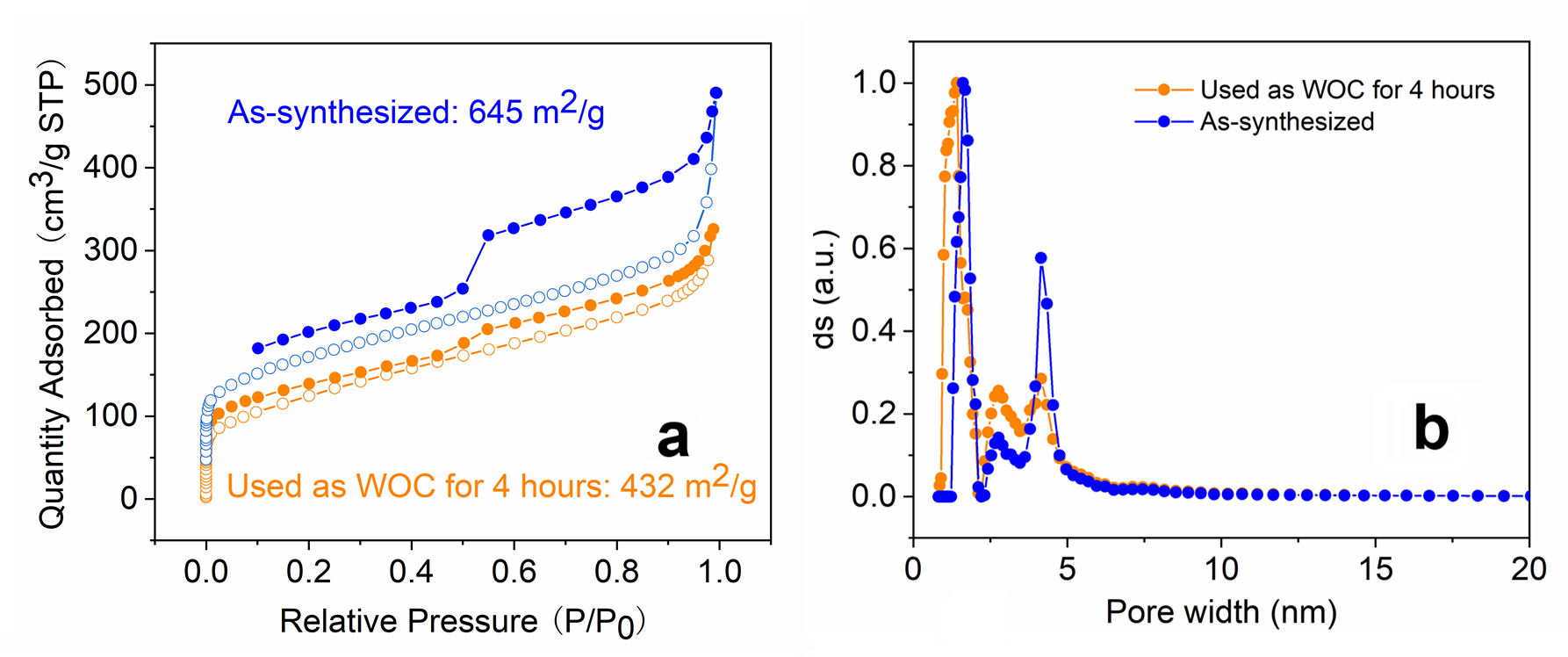


**Figure S22.** (a) 77 K N_2_ adsorption and desorption isotherms and (b) pore size distributions of Tru-COFs (blue) and recycled Tru-COFs (yellow) after used as water oxidation catalyst (WOC) for 4 h. The BET decreased (432 m^2^/g) (Figure S13) compared with the fresh sample, possibly due to a change in the crystallinity.


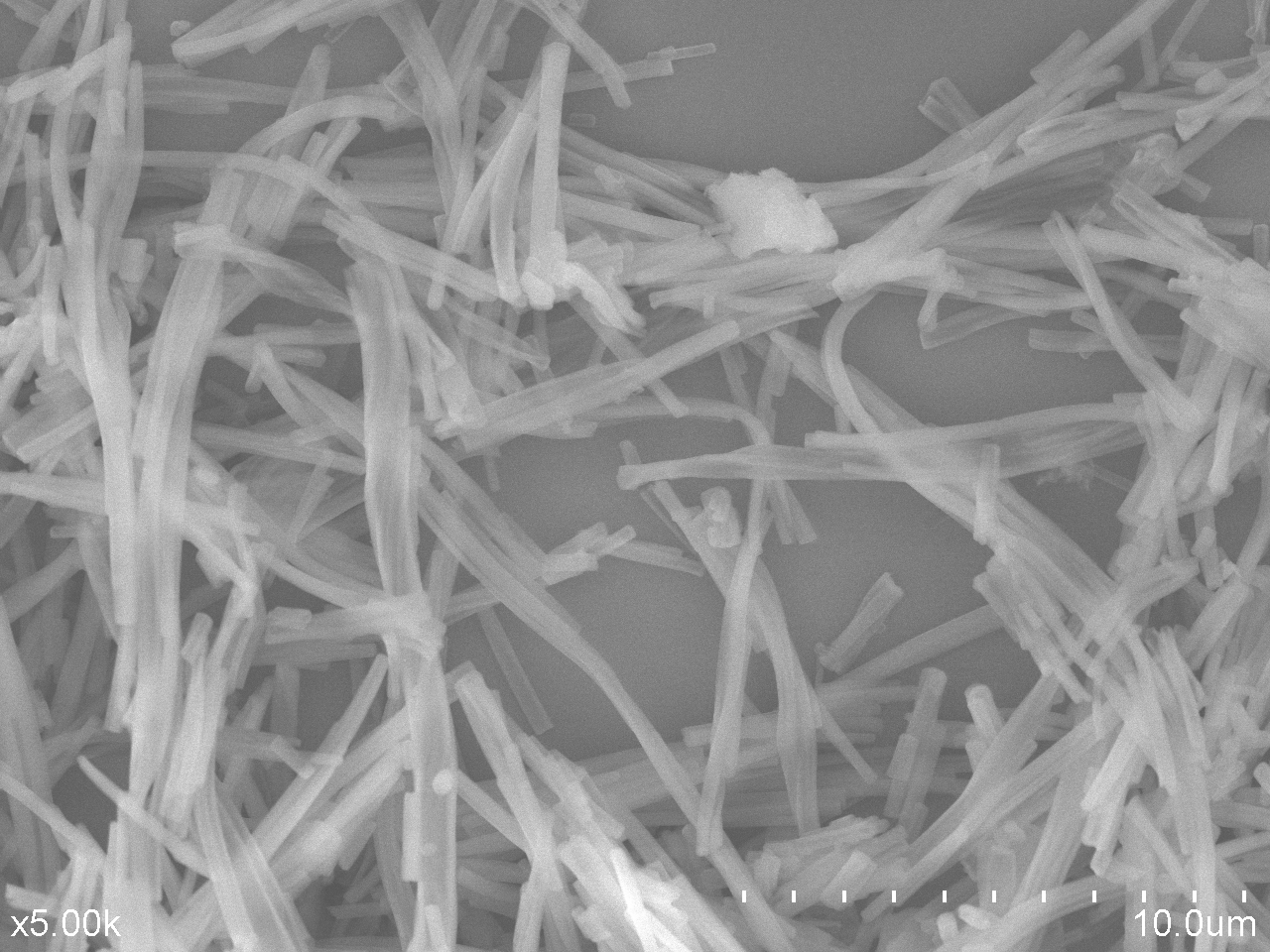


**Figure S23.** SEM image of Tru-COFs (after 4 h of photocatalytic sacrificial oxidation evolution reaction).


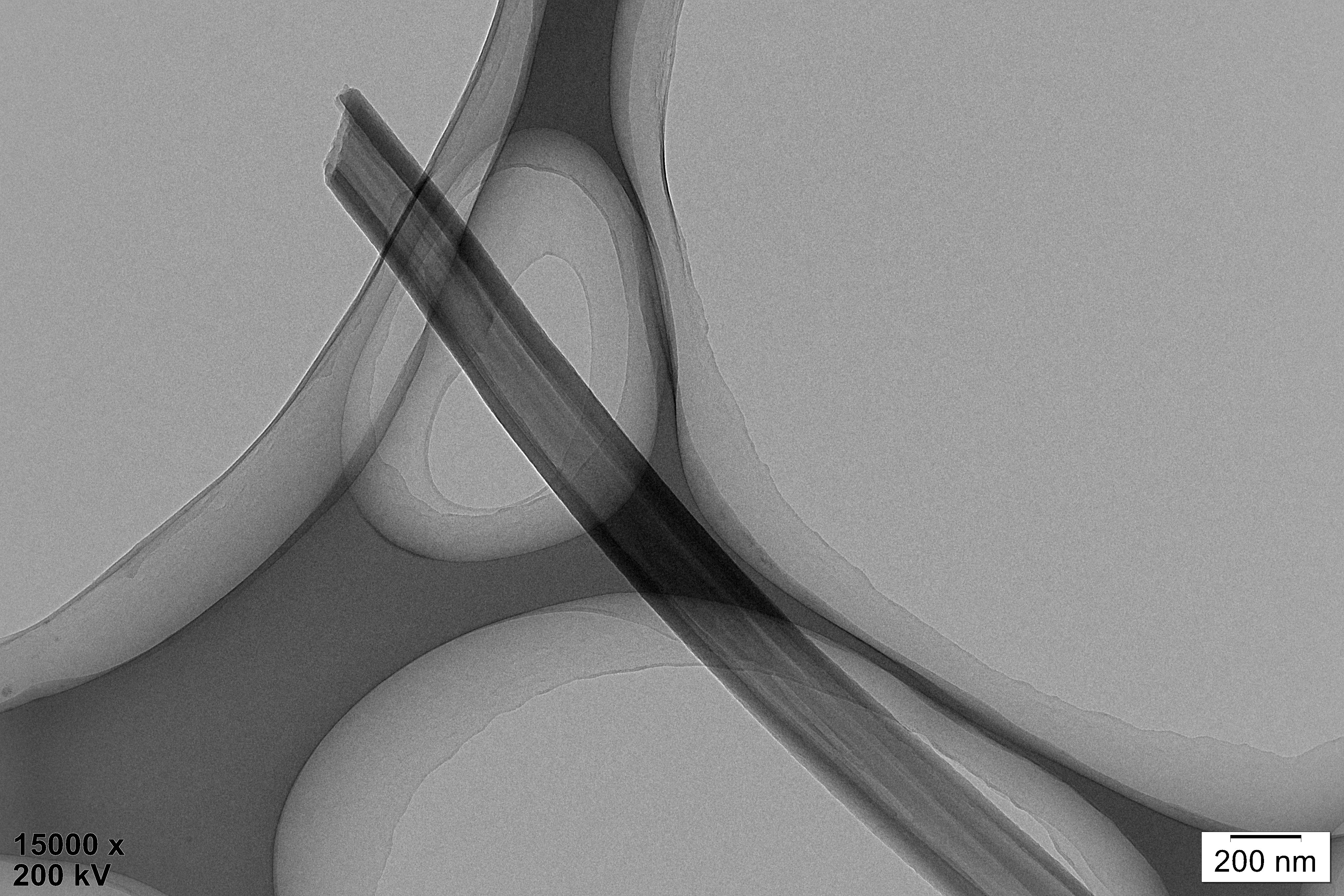


**Figure S24**. TEM image of Tru-COFs (after 4 h of photocatalytic sacrificial oxidation evolution reaction).


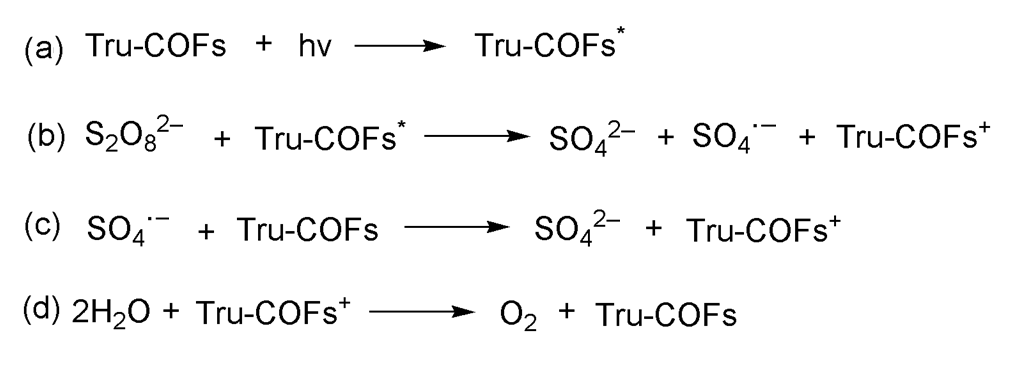


**Figure S25**. Light-driven water oxidation by Tru-COFs with sodium peroxodisulfate as the sacrificial electron acceptor [7,8].


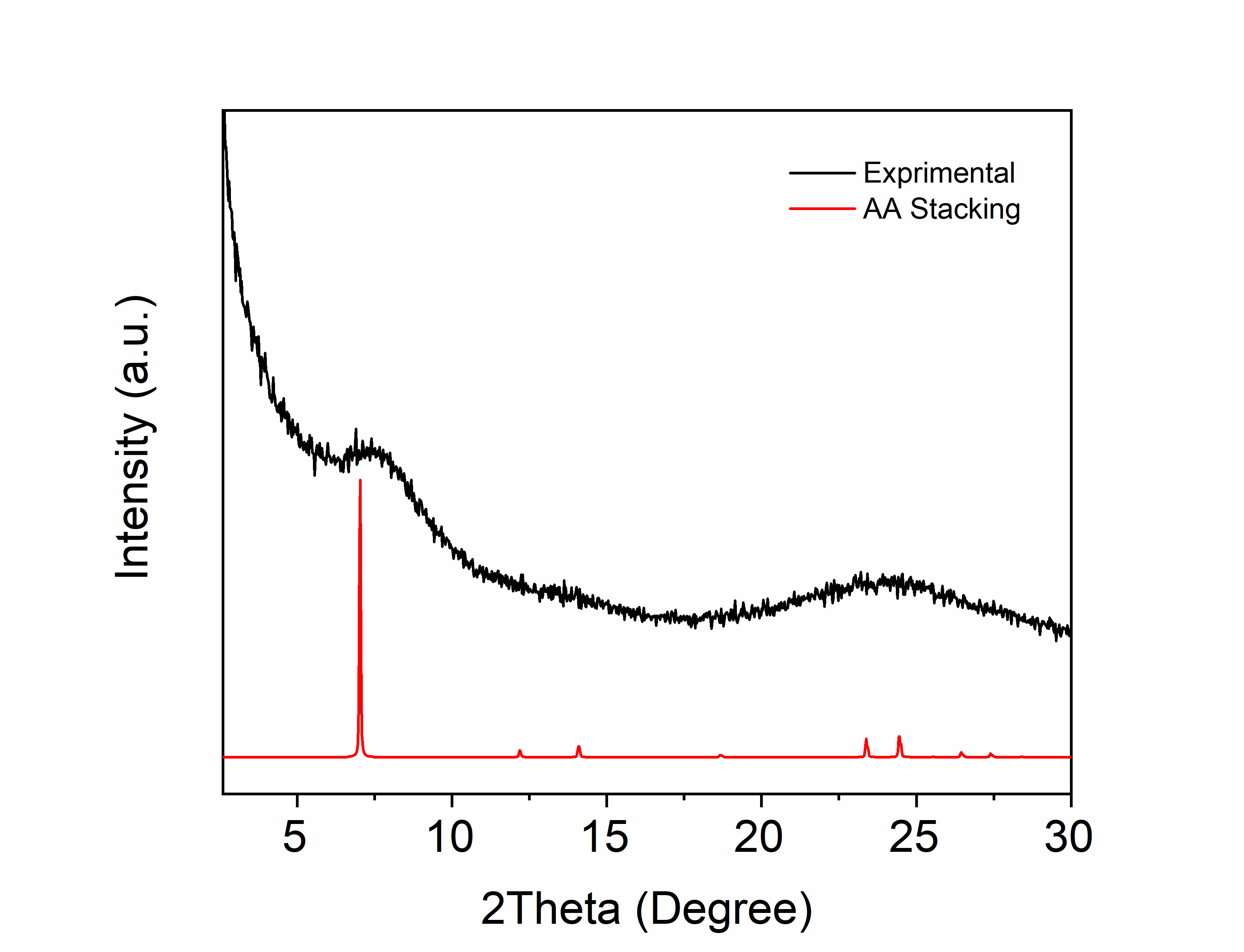


**Figure S26**. Powder X-ray diffraction of Tru-COFs (as-synthesized).


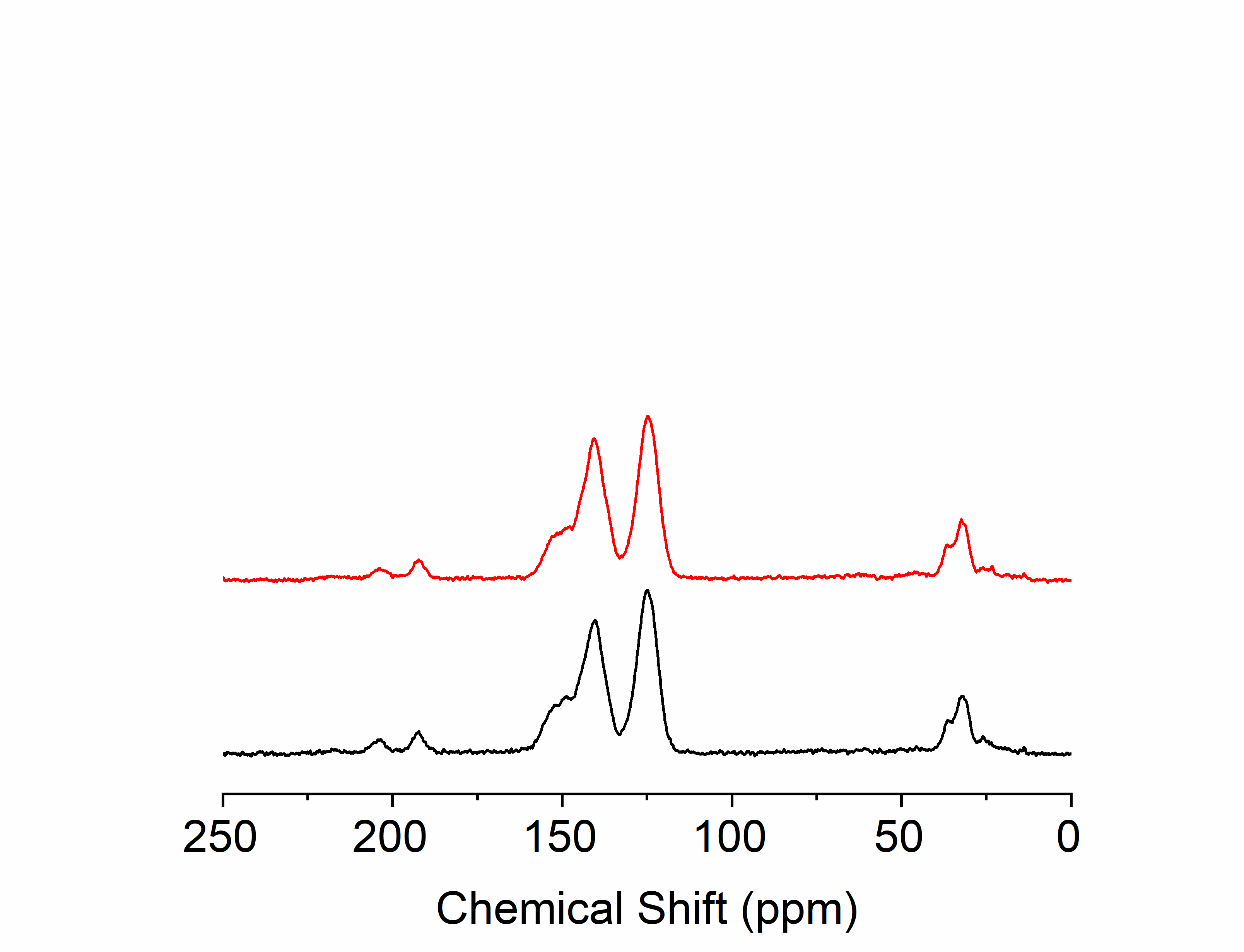


**Figure S27**. Time-dependent growth study of Tru-COFs. Solid state ^13^C CP/MAS NMR (101 MHz) spectra of Tru-COFs after 24 h (black) or 72 h (red) ploymerization.


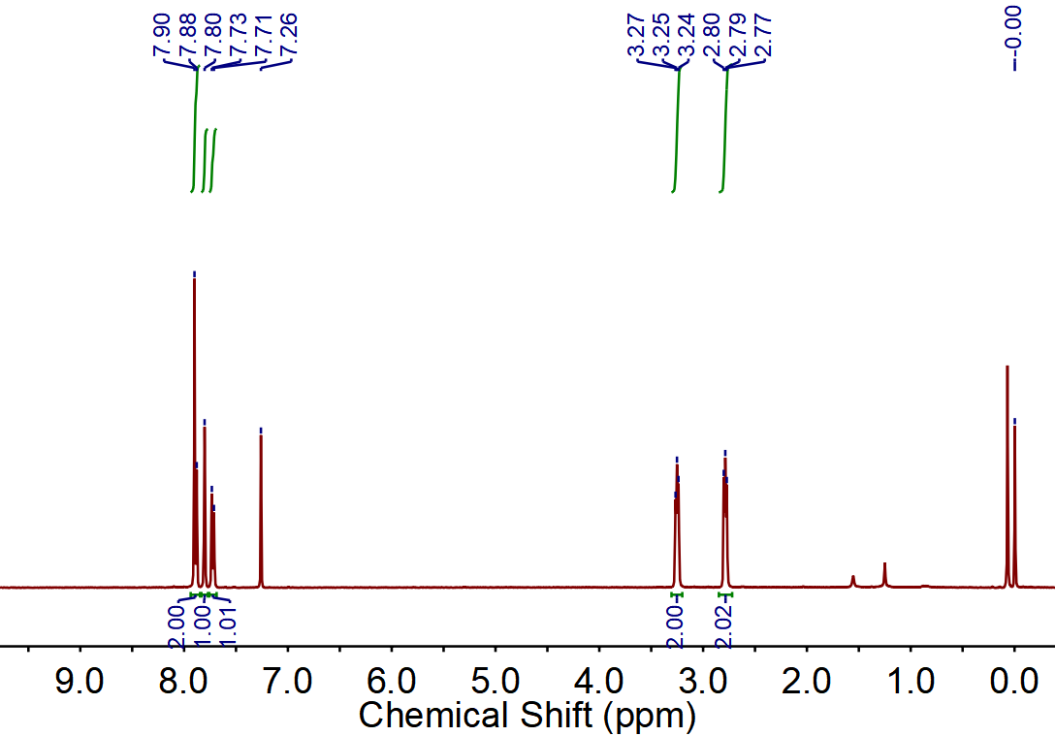


**Figure S28.** ^1^H NMR (400 MHz) spectrum of compound TDB in CDCl_3_.


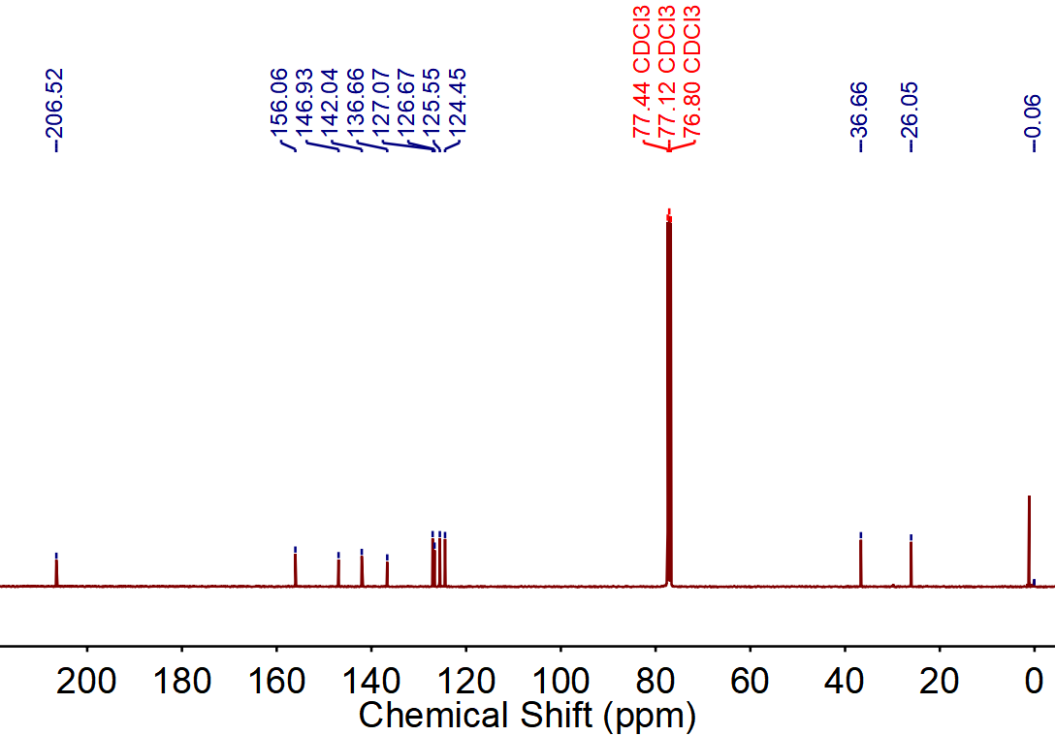


**Figure S29.** ^13^C NMR (101 MHz) spectrum of compound TDB in CDCl_3_.


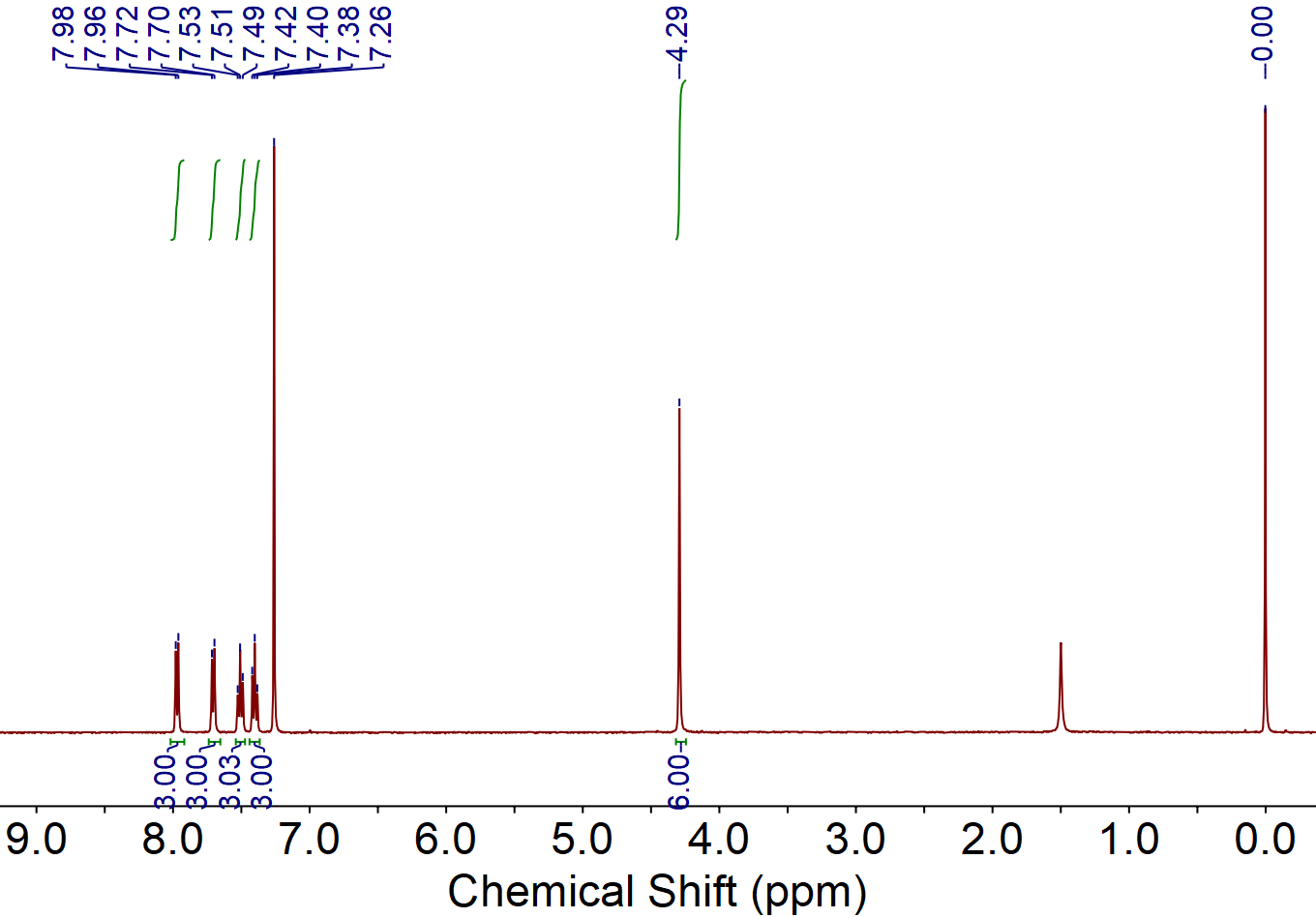


**Figure S30.** ^1^H NMR (400 MHz) spectrum of compound Truxene in CDCl_3_.


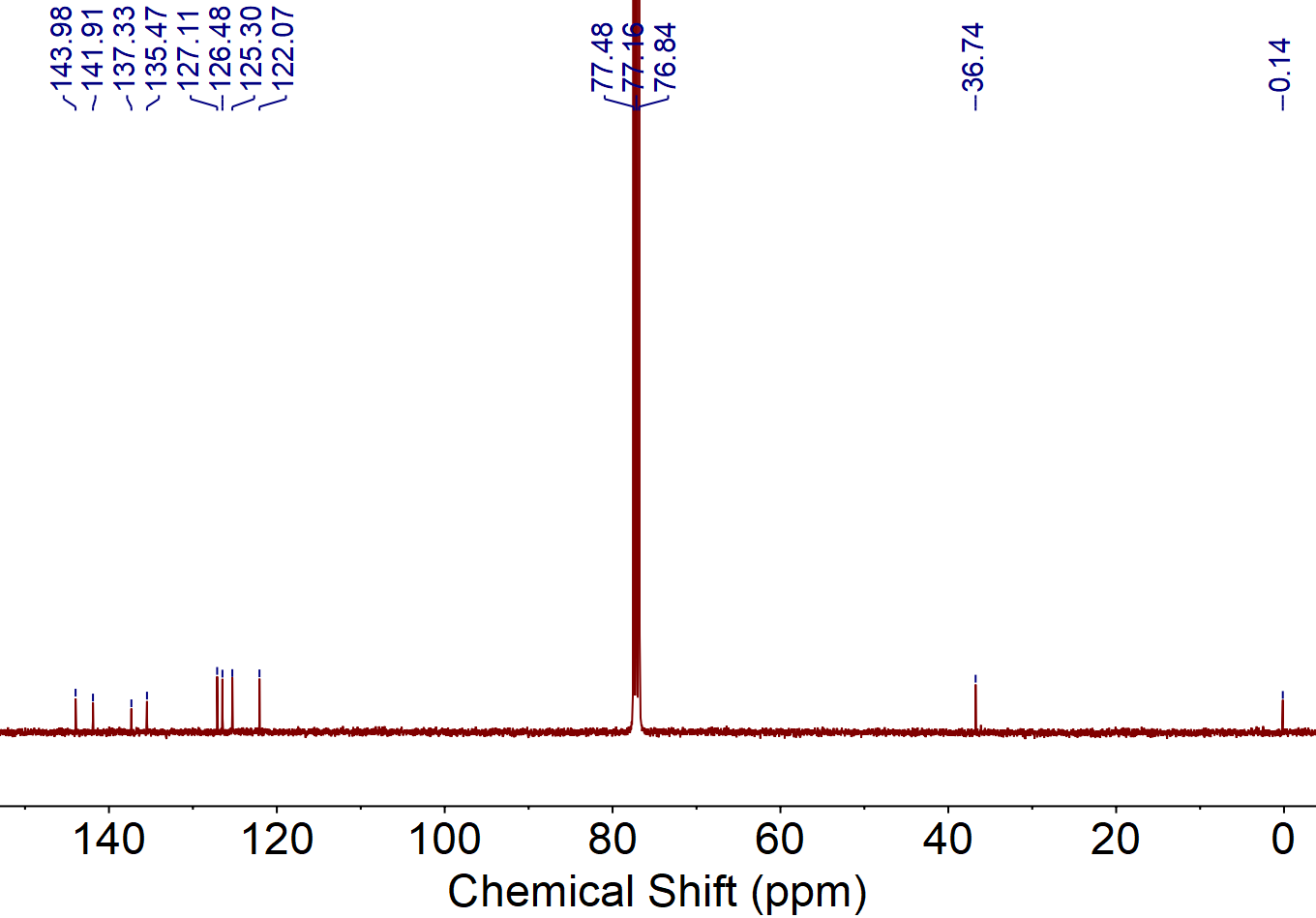


**Figure S31.** ^13^C NMR (101 MHz) spectrum of compound Truxene in CDCl_3_.


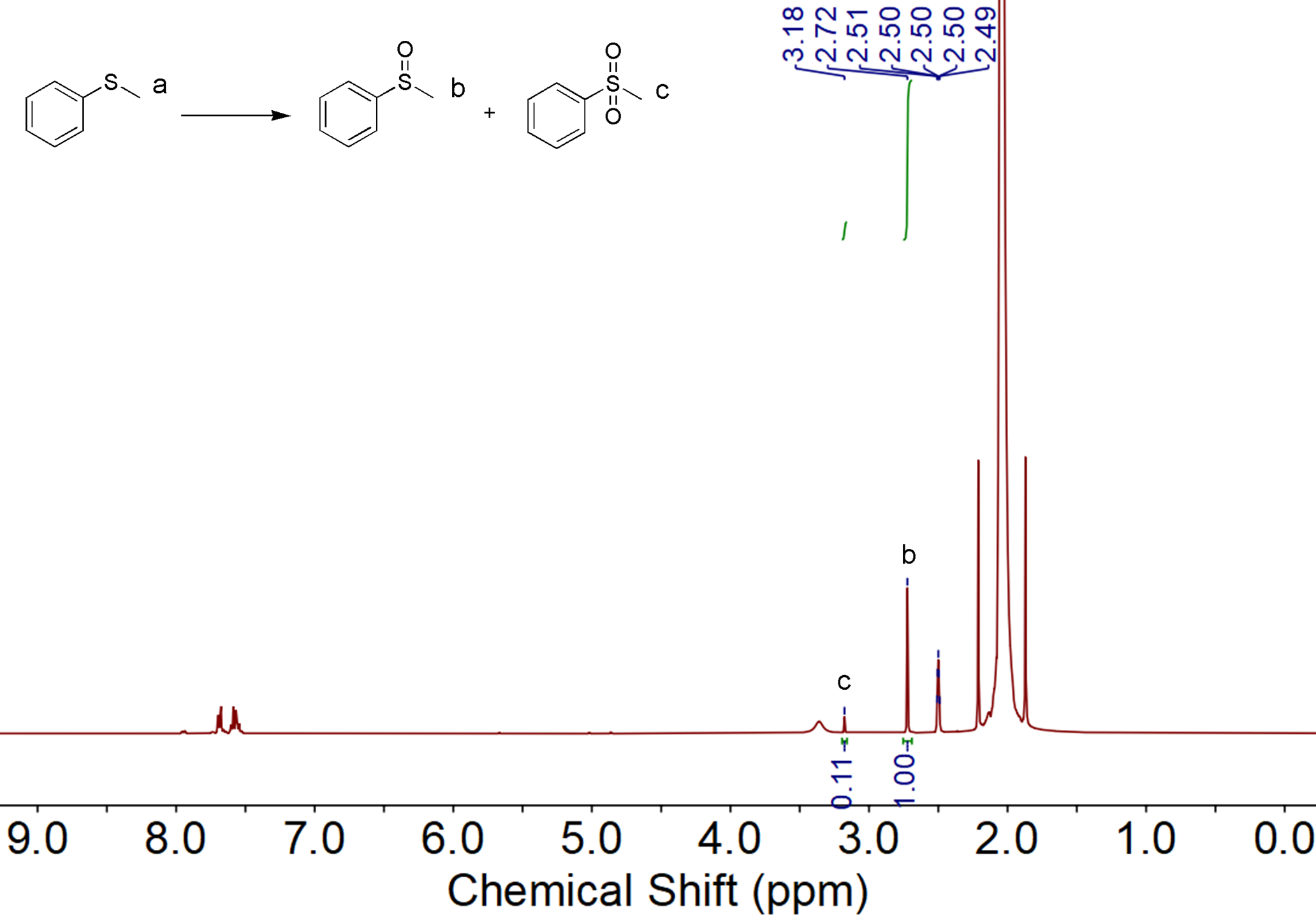


**Figure S32.** ^1^H NMR (400 MHz, DMSO-d6) spectrum for the oxidation of thioanisole catalyzed by Tru-COFs at 4 h (after the 1st cycle of the reaction).


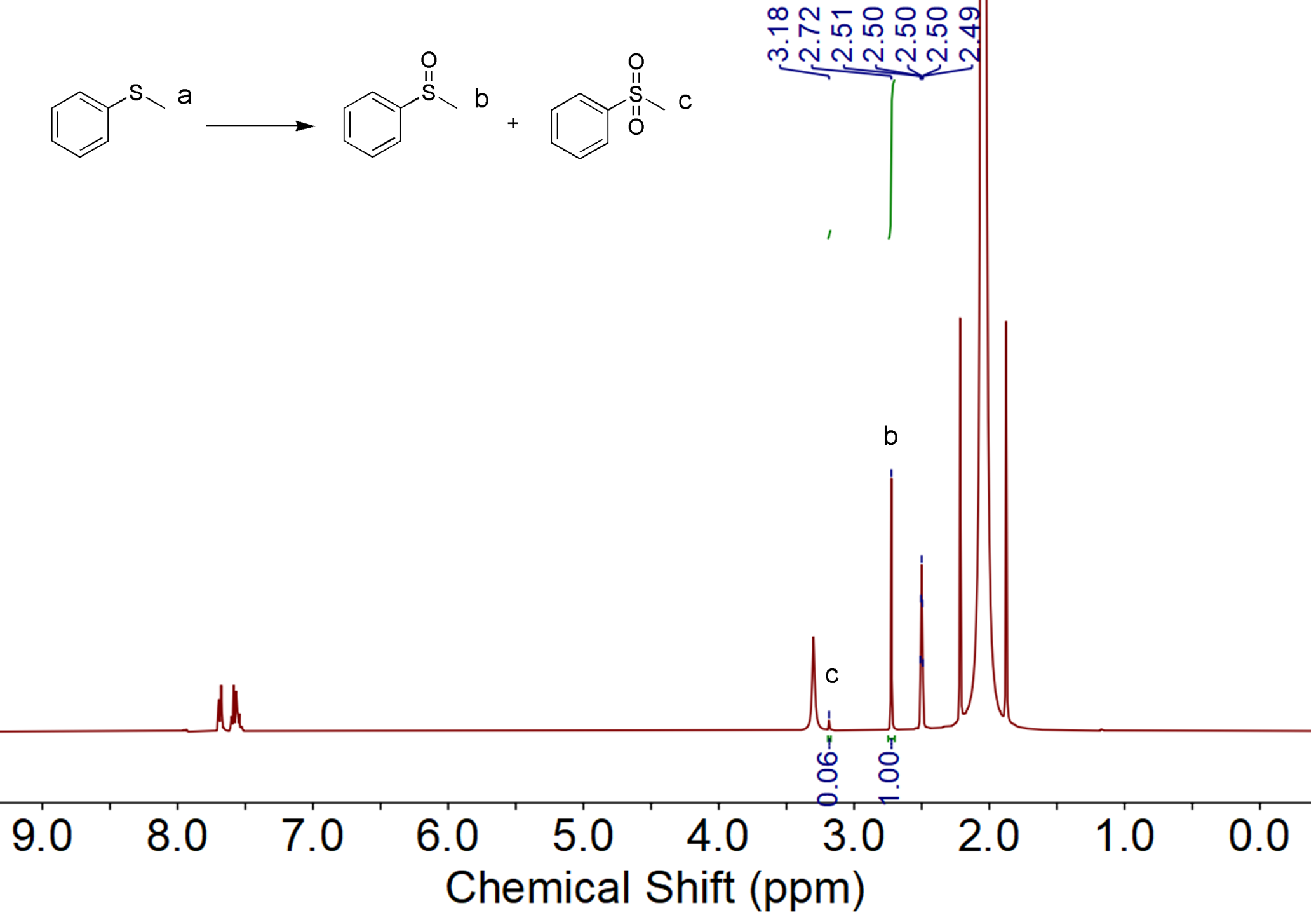


**Figure S33.** ^1^H NMR (400 MHz, DMSO-d6) spectrum for the oxidation of thioanisole catalyzed by Tru-COFs at 4 h (after the 3rd cycle of the reaction).


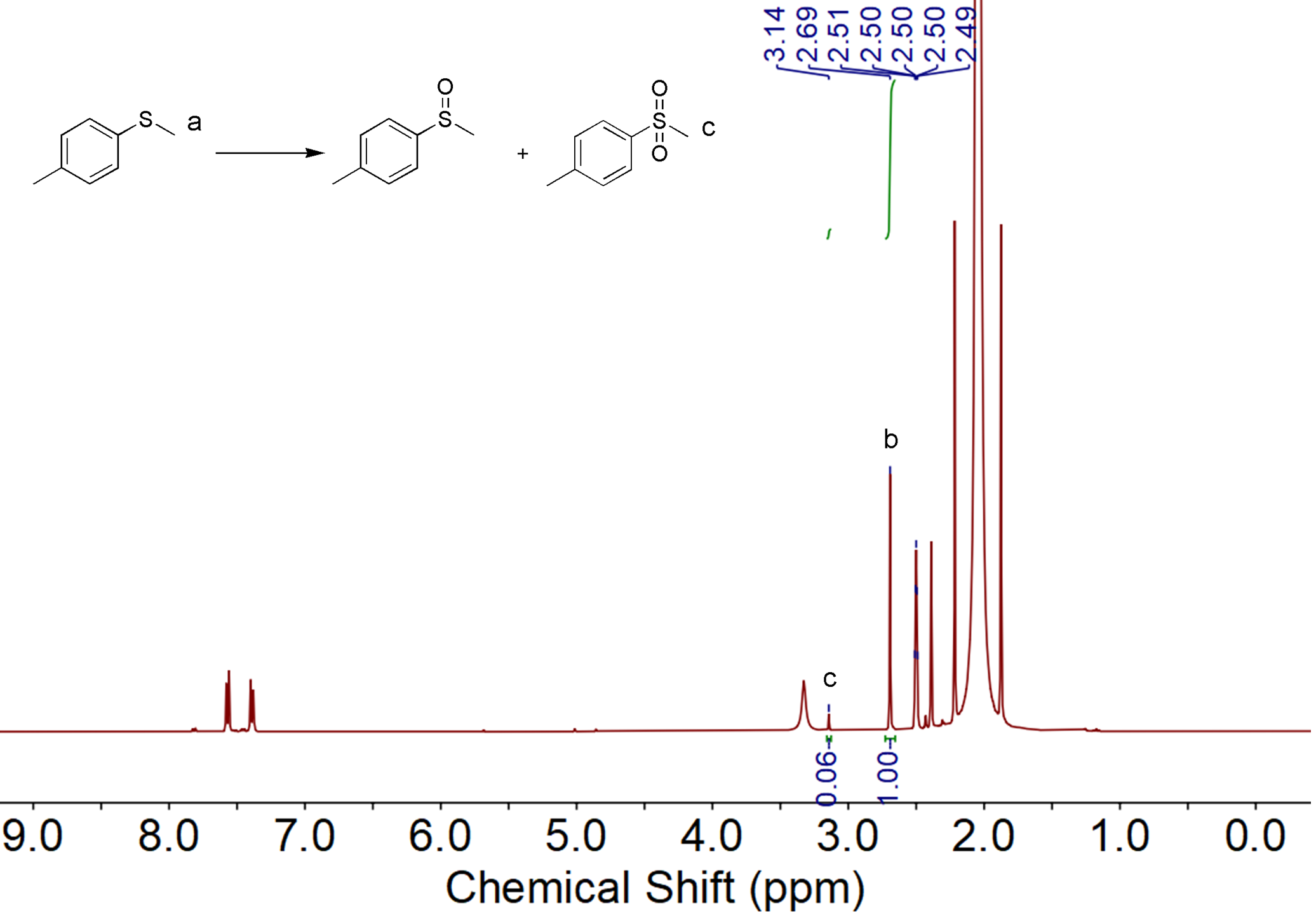


**Figure S34.** ^1^H NMR (400 MHz, DMSO-d6) spectrum for the oxidation of methyl p-tolyl sulfide catalyzed by Tru-COFs at 4 h.


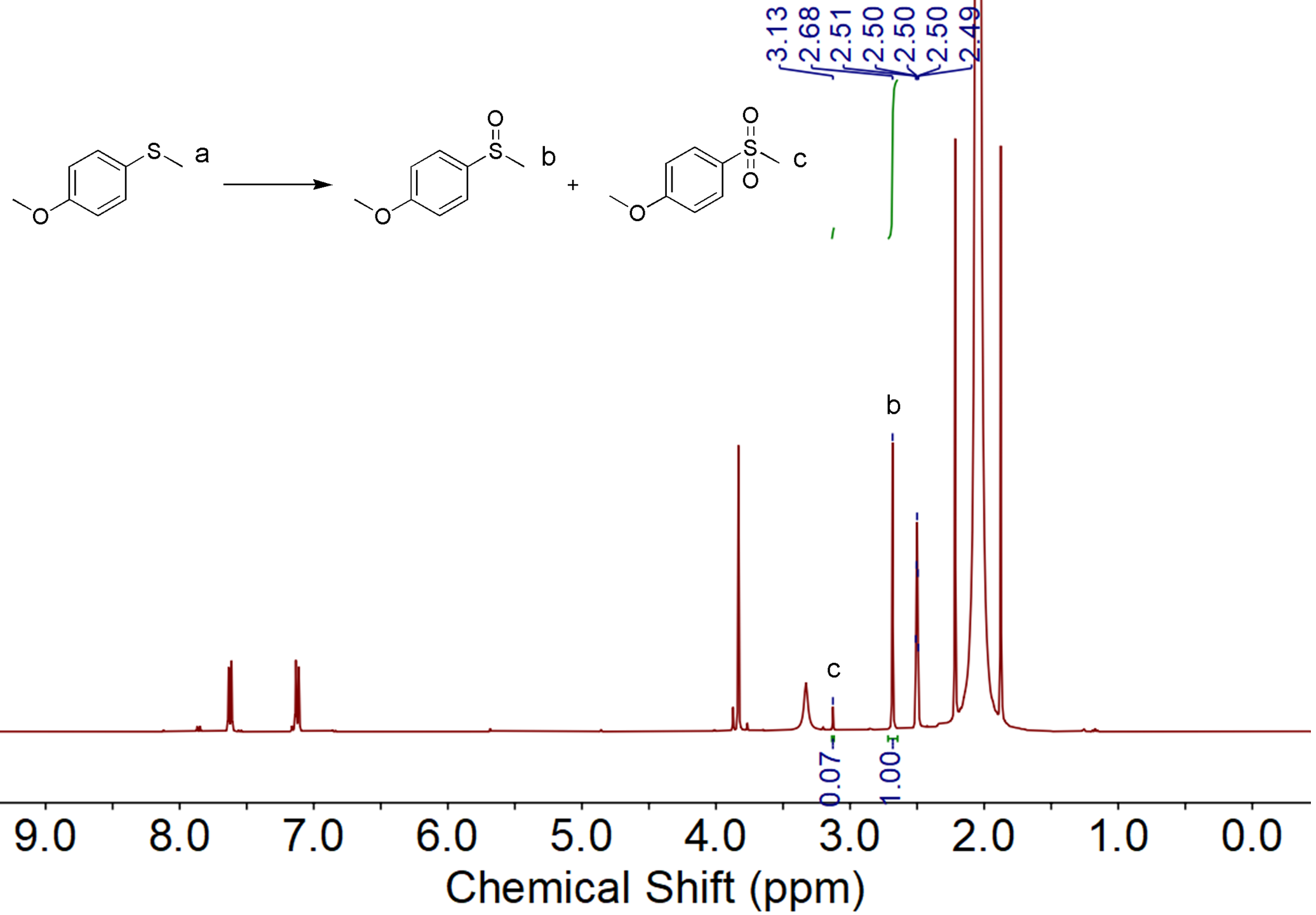


**Figure S35.** ^1^H NMR (400 MHz, DMSO-d6) spectrum for the oxidation of 1-methoxy-4-(methylthio)benzene catalyzed by Tru-COFs at 4 h.


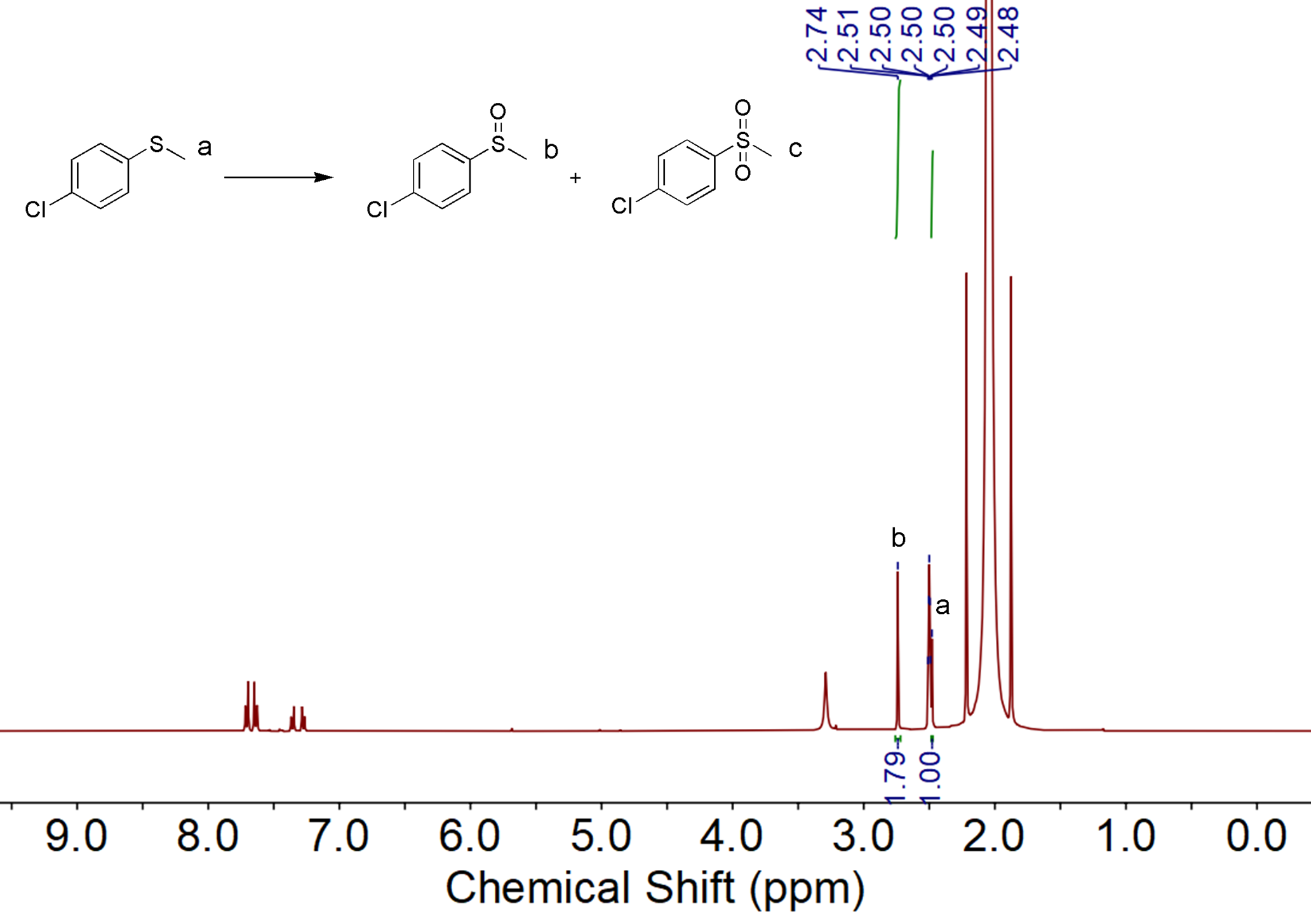


**Figure S36.** ^1^H NMR (400 MHz, DMSO-d6) spectrum for the oxidation of 4-chlorothioanisole catalyzed by Tru-COFs at 4 h.


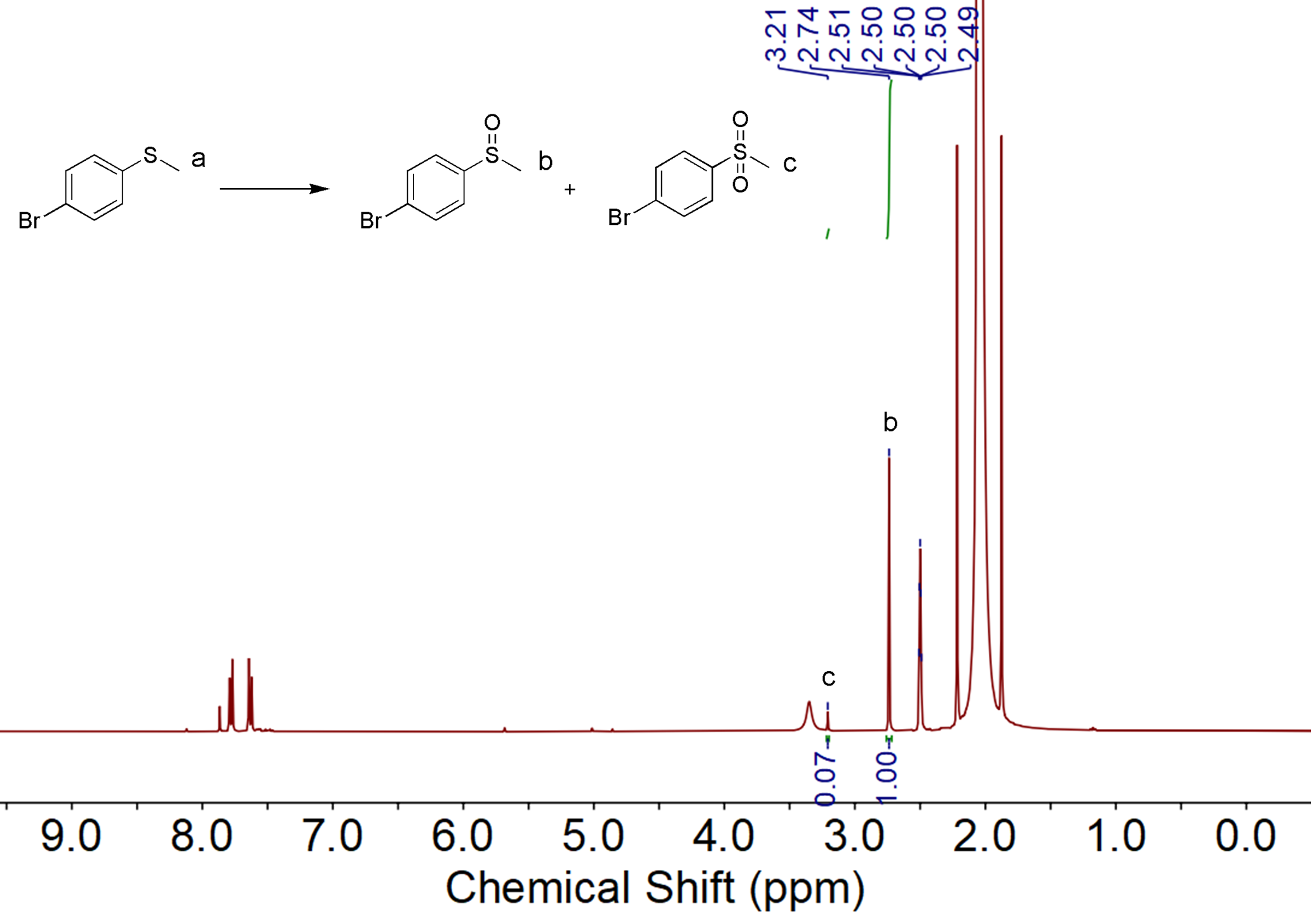


**Figure S37.** ^1^H NMR (400 MHz, DMSO-d6) spectrum for the oxidation of 4-bromothioanisole catalyzed by Tru-COFs at 4 h.


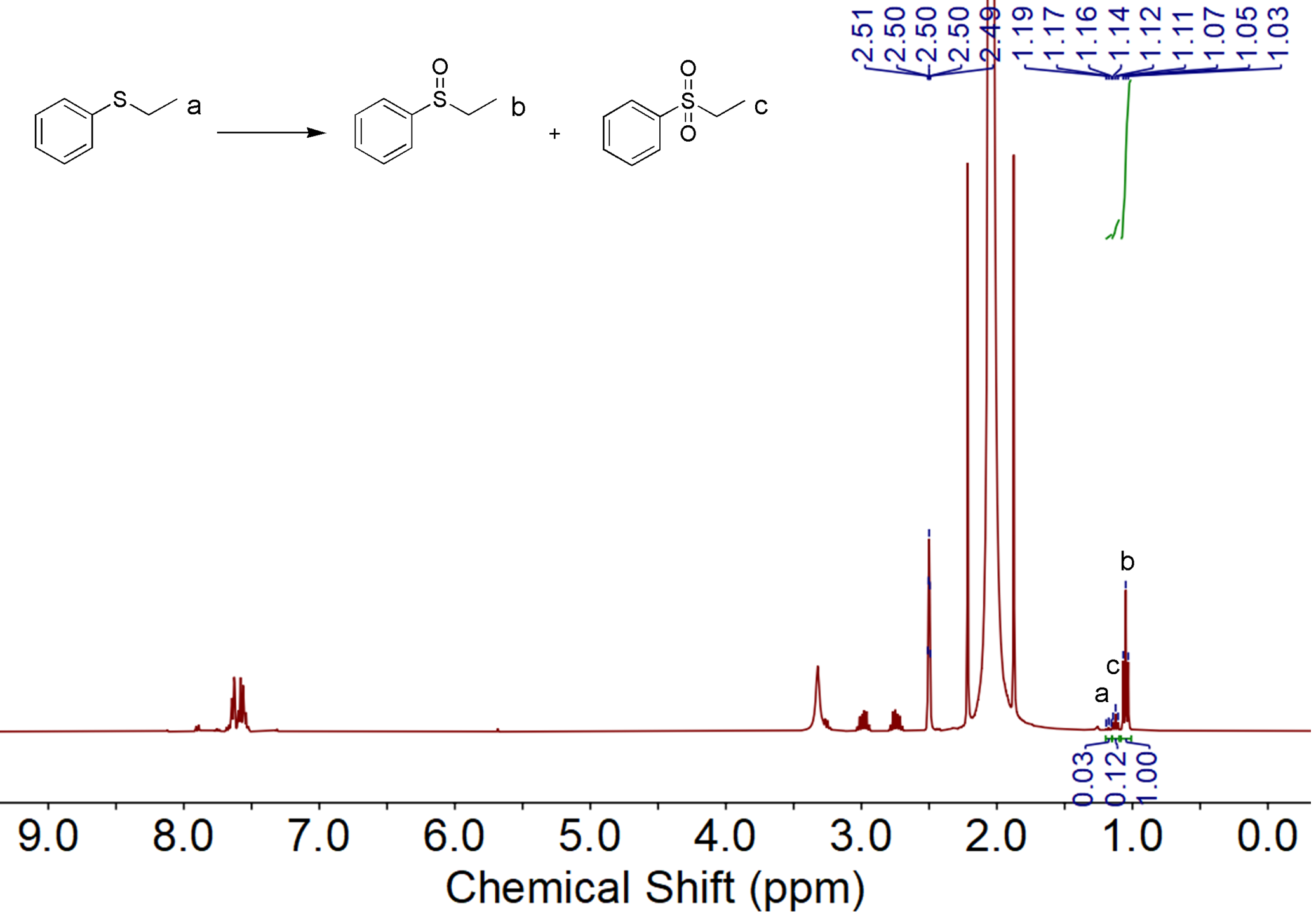


**Figure S38.** ^1^H NMR (400 MHz, DMSO-d6) spectrum for the oxidation of ethyl phenyl sulfide catalyzed by Tru-COFs at 4 h.

**Table S4.** Comparison of the reaction conditions and performances of different catalysts for photocatalytic selective oxidation of thioanisole. *^a^* Conversion (Conv.) and selectivity (Select.) were determined by 1H NMR. *^b^* The yield of benzyl methyl sulfoxide. *^c^* N.D = not detected.

| Catalysts | Conditions | Light source | Time  (h) | Conv.*^a^*  (%) | Select.*^a^*  (%) | Ref. |
| --- | --- | --- | --- | --- | --- | --- |
| Tru-COFs  (5 mg) | Thioanisole (0.25 mmol), CH_3_CN (5 mL), air, 25 ^o^C | 300 W Xe lamp  (λ > 420 nm) | 4 | 100 | 90 | This  work |
| h-LZU1  (10 mg) | thioanisole (0.3 mmol), CH_3_CN (10 mL), air, 30 °C. | visible light source: 300 W Xe lamp  (380 < λ < 780 nm) | 22 | 100 | 92.6 | 2 |
| TiO_2_  (40 mg) | thioanisole (0.3 mmol), triethylamine (redox mediator, 0.03 mmol), CH_3_OH (5 mL), O_2_ (0.1 MPa), 25 °C. | 300 W Xe lamp  (λ > 400 nm) | 10 | 84 | 92 | 3 |
| C_60_/g-C_3_N_4_  (4 wt%, 30 mg) | thioanisole (0.2 mmol), CH_3_OH (5 mL), O_2_, 25 °C. | Xe lamp (1 W cm^-2^, λ > 400 nm) | 6 | 99.9 | 100 | 4 |
| Ir-Zr MOF  (4 mol %, based on  Ir, 14.8 mg) | thioanisole (0.25 mmol), CH_3_CN (4 mL), O_2_, 25 ^o^C | 100 W blue LED | 18 | 79*^b^* | N.D*^c^* | 5 |

**Table S5.** Comparison of the reaction conditions and performances of different catalysts for photocatalytic sacrificial oxygen evolution. *^a^* Turnover number (TON) = The total number of moles of oxygen per mole of precatalyst. The amount of Tru-COFs was calculated based on the smallest repeating unit of the structure. *^b^* (O_2_ evolution) = (The amount of O_2_) / [(The mass of the catalyst) × (chemical reaction time)].

| Catalyst | TON*^a^* | O_2_ evolution*^b^*  (μmol/gh) | Light source | Sacrificial donor | Ref. |
| --- | --- | --- | --- | --- | --- |
| Tru-COFs | 82 | 150 | 300 W Xe lamp  (λ > 360 nm) | Na_2_S_2_O_8_ | This  work |
| Complex 2 | 209 | -- | 500 W Xe lamp  (λ > 400 nm) | Na_2_S_2_O_8_ | 7 |
| [Co_4_(H_2_O)_4_(HPMIDA)_2_(PMIDA)2]^6−^ | 661.5 | -- | 300 W Xe lamp  (λ > 420 nm) | Na_2_S_2_O_8_ | 8 |
| BpCo-COF-1 | -- | 152 | 300 W Xe lamp  (λ > 420 nm) | AgNO_3_ | 9 |
| Salen Co | 854 | -- | LED lamo  (λ > 420 nm) | Na_2_S_2_O_8_ | 10 |

**References**

##### [1] D. N. Bunck, W. R. Dichtel, “[Bulk Synthesis of Exfoliated Two-Dimensional Polymers Using Hydrazone-Linked Covalent Organic Frameworks](https://pubs.acs.org/doi/10.1021/ja408243n),” *J. Am. Chem. Soc*, vol. 135, no. 40, pp. 14952-14955, 2013. Doi: [10.1021/ja408243n](https://doi.org/10.1021/ja408243n).

[2] L. F. Liu, B. X. Zhang, X. N. Tan, D. X. Tan, X. Y. Cheng, B. X. Han and J. L. Zhang, “Improved photocatalytic performance of covalent organic frameworks by nanostructure construction,” *Chem. Commun*, vol. 56, no. 33, pp. 4567**-**4570, 2020. Doi: 10.1039/D0CC00761G.

[3] X. J. Lang, W. Hao, W. R. Leow, S. Z. Li, J. C. Zhao and X. D. Chen, “Tertiary amine mediated aerobic oxidation of sulfides into sulfoxides by visible-light photoredox catalysis on TiO_2_,” *Chem. Sci*, vol. 6, no. 8, 5000-5005, 2015. Doi: 10.1039/C5SC01813G.

[4] X. Chen, K. J. Deng, P. Zhou and Z. H. Zhang, “Metal- and Additive-Free Oxidation of Sulfides into Sulfoxides by Fullerene-Modified Carbon Nitride with Visible-Light Illumination”, *ChemSusChem*, vol. 11, no. 14, 2444-2452, 2018. Doi: 10.1002/cssc.201800450.

[5] L.-Q. Wei and B.-H. Ye, “Cyclometalated Ir–Zr Metal–Organic Frameworks as Recyclable Visible-Light Photocatalysts for Sulfide Oxidation into Sulfoxide in Water,” *ACS Appl. Mater. Interfaces,* vol. 11, no. 44, pp. 41448-41457, 2019. Doi: 10.1021/acsami.9b15646.

[6] S. Y. Liu， M. Tian, X. B. Bu, H. T. Tian, X. B. Yang, “Covalent Organic Frameworks toward Diverse Photocatalytic Aerobic Oxidations,” *Chem. Eur.* *J*, vol. 27, no. 28, pp. 7738-7744, 2021. Doi: 10.1002/chem.202100398.

[7] Q. Xu, H. Li, L. Chi, L. G. Zhang, Z. Wan, Y. Ding, J. D. Wang, “Identification of homogeneous [Co_4_(H_2_O)_4_(HPMIDA)_2_(PMIDA)_2_]^6−^ as an effective molecular-light-driven water oxidation catalyst,” *Appl. Catal. B: Environ*, vol. 202, pp. 397-403, 2017. Doi: 10.1016/j.apcatb.2016.09.056.

[8] L. Wang, M. Mirmohades, A. Brown, L. Duan, F. S. Li, Q. Daniel, R. Lomoth, L. C. Sun, L. Hammarström, “Sensitizer-Catalyst Assemblies for Water Oxidation,” *Inorg. Chem*. vol. 54, pp. 2742-2751, 2015. Doi: [10.1021/ic502915r](https://doi.org/10.1021/ic502915r).

[9] J. Chen, X. P. Tao, C. Z. Li, Y. H. Ma, L. Tao, D. Y. Zheng, J. F. Zhu, H. Li, R. G. Li, Q. H. Yang, “Synthesis of bipyridine-based covalent organic frameworks for visible-light-driven photocatalytic water oxidation,” *Appl. Cataly. B: Environ.* vol. 262, pp. 11827, 2020. Doi: [10.1016/j.apcatb.2019.118271](https://doi.org/10.1016/j.apcatb.2019.118271).

[10] S. Fu, Y. Liu, Y. Ding, X. Du, F. Song, R. Xiang, B. Ma, “A mononuclear cobalt complex with an organic ligand acting as a precatalyst for efficient visible light-driven water oxidation,” *Chem. Commu*, vol. 50, pp. 2167-2169, 2014. Doi: [10.1039/c3cc48059c](https://www.x-mol.com/paperRedirect/2223227).
